# Impaired Cdc20 signaling promotes senescence in normal cells and apoptosis in non–small cell lung cancer cells

**DOI:** 10.1016/j.jbc.2022.102405

**Published:** 2022-08-19

**Authors:** Daniela Volonte, Morgan Sedorovitz, Ferruccio Galbiati

**Affiliations:** Department of Pharmacology & Chemical Biology, University of Pittsburgh School of Medicine, Pittsburgh, Pennsylvania, USA

**Keywords:** senescence, Cdc20, APC/C, securin, apoptosis, CSE, cigarette smoke extract, DAPI, 4,6-diamidino-2-phenylindole, DDR, DNA damage response, MEF, mouse embryonic fibroblast, MTT, 3-(4,5-dimethylthiazol-2-yl)-2,5-diphenyltetrazolium bromide, NHBE, normal human bronchial epithelial, NSCLC, non–small cell lung cancer, SA, senescence-associated, SAC, spindle assembly checkpoint, SIPS, stress-induced premature senescence

## Abstract

Cellular senescence is a form of irreversible growth arrest that cancer cells evade. The cell division cycle protein 20 homolog (Cdc20) is a positive regulator of cell division, but how its dysregulation may relate to senescence is unclear. Here, we find that Cdc20 mRNA and protein expression are downregulated in stress-induced premature senescent lung fibroblasts in a p53-dependent manner. Either Cdc20 downregulation or inhibition of anaphase-promoting complex/cyclosome (APC/C) is sufficient to induce premature senescence in lung fibroblasts, while APC/C activation inhibits stress-induced premature senescence. Mechanistically, we show both Cdc20 downregulation and APC/C inhibition induce premature senescence through glycogen synthase kinase (GSK)-3β–mediated phosphorylation and downregulation of securin expression. Interestingly, we determined Cdc20 expression is upregulated in human lung adenocarcinoma. We find that downregulation of Cdc20 in non–small cell lung cancer (NSCLC) cells is sufficient to inhibit cell proliferation and growth in soft agar and to promote apoptosis, but not senescence, in a manner dependent on downregulation of securin following GSK-3β-mediated securin phosphorylation. Similarly, we demonstrate securin expression is downregulated and cell viability is inhibited in NSCLC cells following inhibition of APC/C. Furthermore, we show chemotherapeutic drugs downregulate both Cdc20 and securin protein expression in NSCLC cells. Either Cdc20 downregulation by siRNA or APC/C inhibition sensitize, while securin overexpression inhibits, chemotherapeutic drug-induced NSCLC cell death. Together, our findings provide evidence that Cdc20/APC/C/securin-dependent signaling is a key regulator of cell survival, and its disruption promotes premature senescence in normal lung cells and induces apoptosis in lung cancer cells that have bypassed the senescence barrier.

Cellular senescence is an irreversible form of cell cycle arrest (1, 2, 3, 4). Cellular senescence can be divided into two main categories: replicative senescence and stress-induced premature senescence (SIPS). Replicative senescence is dependent on the number of divisions the cell has completed. Senescence can be accelerated by a number of stressful stimuli such as oncogene activation, DNA damage, cytotoxic drugs, and oxidative stress (5, 6, 7, 8, 9). This type of senescence is referred to as SIPS. Senescent cells are characterized by enlarged and flat cell morphology and by well-defined molecular changes, including increased β-galactosidase activity at pH 6, enhanced p53 activity, and elevated p21^Waf1/Cip1^ and p16 protein expression (5, 6, 7, 8, 9, 10, 11). Senescent cells are also characterized by a chronic DNA damage response (DDR) with elevated γ-H2A.X expression and DNA damage foci (10) and senescence-associated heterochromatin foci (11) formation. In addition, senescent cells secrete a plethora of cytokines, growth factors, and proteases, known as the senescence-associated secretory phenotype (12, 13, 14, 15, 16, 17, 18, 19, 20). Senescent cells accumulate in tissues over time and contribute to both aging and the development of age-associated diseases. Senescent cells have antagonistic pleiotropic roles in cancer. Since senescent cells are unable to proliferate, cellular senescence is a powerful tumor suppressor mechanism. Consistent with an anti-tumorigenic role of senescence, senescent cells accumulate at sites of premalignant lesions in mice and humans and are absent or rare in malignant tumors (8, 21, 22, 23, 24, 25, 26, 27, 28, 29, 30). However, accumulation of senescent cells during aging can promote cancer cell growth given their ability to release factors that stimulate cell proliferation. The signaling pathways and molecular mechanisms that regulate the acquisition of a senescent phenotype are therefore of upmost importance in cancer biology.

Cdc20 is a key positive regulator of cell division. Proper chromosome attachment to the mitotic spindle is guaranteed by the spindle assembly checkpoint (SAC), which prevents anaphase onset (31). The SAC inhibits Cdc20. Once proper chromosome alignment has been achieved (32, 33, 34), the SAC is inactivated, leading to the Cdc20-mediated activation of the anaphase-promoting complex/cyclosome (APC/C). APC/C is an E3 ubiquitin ligase that triggers anaphase and mitotic exit by promoting the degradation of securin and cyclin B1 (35, 36, 37, 38). Degradation of securin releases the protease separase, which promotes degradation of the cohesion complex allowing sister chromatids to move to opposite spindle poles. Degradation of cyclin B1 inactivates mitotic cyclic–dependent kinase (Cdk1) complexes, which promotes the exit from mitosis. Impaired Cdc20-APC/C function delays mitotic exit and induces a chronic DDR signaling. Whether dysregulation of Cdc20 and APC/C is functionally linked to the development of senescence is largely unknown.

Evidence indicates that Cdc20 plays an important role in tumorigenesis. Overexpression of Cdc20 has been observed in a variety of human tumors, including lung, gastric, and breast cancer, where its activity has been associated with tumor initiation, maintenance, and growth (39, 40, 41, 42). The genetic ablation of endogenous Cdc20 has been shown to inhibit tumorigenesis in a skin-tumor mouse model (43). Similarly, studies show that securin expression has prognostic values in esophageal, thyroid, hepatocellular, and colorectal carcinomas, malignant melanoma, and glioma (44, 45, 46, 47, 48, 49). Apoptosis is an anti-tumorigenic event. Tumor cells need to escape apoptosis in order to generate a relevant tumor mass. Most chemotherapeutic drugs inhibit cancer growth by promoting apoptosis of cancer cells. Although chemotherapeutic drugs are effective means in cancer therapy, their use can be limited by undesirable toxicity. Moreover, defective apoptotic programs may contribute to treatment failure. Thus, studies are warrant to better understand the molecular mechanisms through which chemotherapeutic drugs induce apoptotic cell death of cancer cells in order to develop improved therapeutic options for cancer patients.

In the present study, we find that loss of Cdc20-APC/C–mediated signaling has different functional outcomes in normal and cancer cells. We provide evidence that either loss of Cdc20 expression or inhibition of APC/C activity promotes senescence in normal human lung fibroblasts while it causes apoptosis in non–small cell lung cancer (NSCLC) cells through a mechanism that requires loss of securin expression. Together, these findings provide novel insights into the signaling mechanisms that control the development of senescence and apoptosis, two cellular events that are relevant to the fields of aging, age-related disease, and cancer.

## Results

### Cdc20 expression is downregulated in stress-induced and replicative senescent fibroblasts in a p53-dependent manner

**Figure 1:**
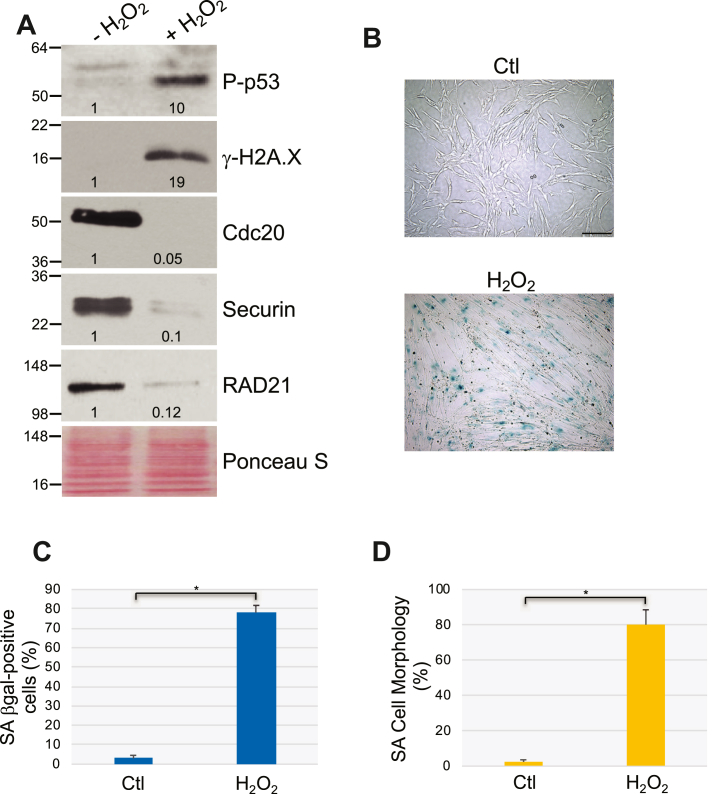
**Cdc20 and securin protein expression is downregulated in oxidative stress–induced senescent WI-38 fibroblasts.***A*, human diploid WI-38 fibroblasts were treated with sublethal hydrogen peroxide (450 μM) for 2 h. Cells were washed with PBS and recovered in complete medium for 14 days. Cells were collected and cell lysates were subjected to immunoblot analysis using protein-specific antibody probes. Untreated cells were used as control. Ponceau S staining shows equal total protein loading. Quantification of protein band intensity is shown at the bottom of each blot. *B*–*D*, premature senescence was induced in WI-38 cells as described in (*A*). Cells were then stained to detect senescence-associated β-galactosidase activity. Representative images are shown in (*B*), quantification is shown in (*C*). The scale bar represents 100 μm. The percentage of cells possessing enlarged and flat morphology (senescence-associated (SA) cell morphology) is shown in (*D*). Values in (*C*) and (*D*) represent means ± SEM; statistical comparisons were made using the student’s *t* test. ∗*p* < 0.001.

To begin investigating the role of Cdc20 in cellular senescence, we determined how Cdc20 protein expression changed in cells that were induced to premature senesce by external stressors. To this end, we exposed WI-38 human diploid lung fibroblasts to oxidative stress by treating the cells to sublethal levels of hydrogen peroxide (H_2_O_2_). We find that sublethal oxidative stress induced senescence in WI-38 cells, as shown by immunoblotting analysis using antibodies specific for the senescence markers phospho-p53 and γ-H2A.X (Fig. 1*A*) and by quantification of cells that are positive for senescence-associated β-galactosidase (SA-β-gal) activity (Fig. 1, *B* and *C*) and senescence-associated cell morphology (Fig. 1*D*). Interestingly, while Cdc20 protein expression was detectible in growing WI-38 cells, Cdc20 protein expression was reduced to almost undetectable levels in senescent WI-38 cells (Fig. 1*A*). Consistent with these data, we find that treatment with either UV-C light or cigarette smoke extracts (CSEs) also induced premature senescence (Fig. S1*A*-*D* and S2*A*-*D*, respectively) and drastically reduced Cdc20 protein expression in WI-38 cells, as compared with nonsenescent control cells (Figs. S1*A* and S2*A*). Thus, Cdc20 is downregulated in premature senescent fibroblasts.

**Figure 2:**
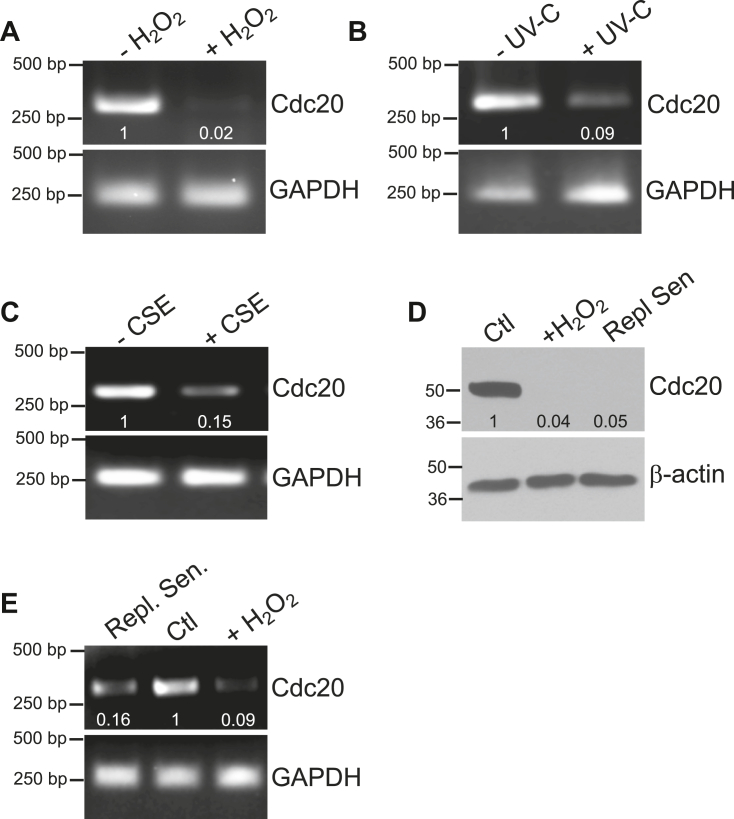
**Cdc20 mRNA is downregulated in stress-induced and replicative senescent WI-38 fibroblasts.***A*, WI-38 fibroblasts were treated with sublethal hydrogen peroxide (450 μM) for 2 h to induce premature senescence. Cells were washed with PBS and recovered in complete medium for 14 days. Cells were collected and the expression level of Cdc20 mRNA was determined by RT-PCR using primers specific for Cdc20. Untreated cells were used as control. Expression of GAPDH was detected as internal control. *B*, human WI-38 fibroblasts were treated with sublethal UV-C light (10 J/m^2^) to induce premature senescence. Cells were then recovered in complete medium for 14 days. Cells were collected and the expression level of Cdc20 mRNA was determined by RT-PCR using primers specific for Cdc20. Untreated cells were used as control. Expression of GAPDH was detected as internal control. *C*, WI-38 cells were treated with 2% cigarette smoke extracts (CSEs) for 14 days to induce premature senescence. Cells were collected and the expression level of Cdc20 mRNA was determined by RT-PCR using primers specific for Cdc20. Untreated cells were used as control. Expression of GAPDH was detected as internal control. *D*, lysates from nonsenescent (Ctl) and replicative senescent human WI-38 cells were subjected to immunoblotting analysis using anti-Cdc20 IgGs. Lysate from oxidative stress–induced premature senescent WI-38 cells was used as positive control. Anti-β-actin IgGs were used to show equal total protein loading. Quantification of protein band intensity is shown at the bottom of the blot. *E*, expression of Cdc20 mRNA in nonsenescent (Ctl) and replicative senescent WI-38 fibroblasts was determined by RT-PCR using primers specific for Cdc20. Cdc20 mRNA expression in oxidative stress–induced senescent WI-38 cells served as positive control. Expression of GAPDH was detected as internal control. Quantification of mRNA band intensity is shown at the bottom of each blot.

To determine whether the severe downregulation of Cdc20 protein expression observed in premature senescent WI-38 fibroblasts occurred at the transcriptional or translational level, we assessed Cdc20 mRNA expression by RT-PCR in WI-38 cells before and after induction of premature senescence with oxidative stress, UV-C light, and CSEs. We find that, similarly to Cdc20 protein expression, these senescence-inducing stimuli blunted Cdc20 mRNA expression to virtually undetectable levels (Fig. 2, *A*–*C*). In addition, we find that treatment with the proteosomal inhibitors MG-132, chloroquine, or ammonium chloride did not prevent Cdc20 protein downregulation induced by oxidative stress in senescent WI-38 fibroblasts (Fig. S3*A*). These results indicate that downregulation of Cdc20 in premature senescent human diploid fibroblasts occurs through a mechanism that inhibits Cdc20 gene transcription.

Importantly, downregulation of Cdc20 was not limited to premature senescent cells but occurred also during replicative senescence. In fact, similarly to stress-induced premature senescent WI-38 cells, Cdc20 protein (Fig. 2*D*) and mRNA (Fig. 2*E*) expression was dramatically reduced in replicative senescent WI-38 fibroblasts, as compared to their nonsenescent counterparts.

**Figure 3:**
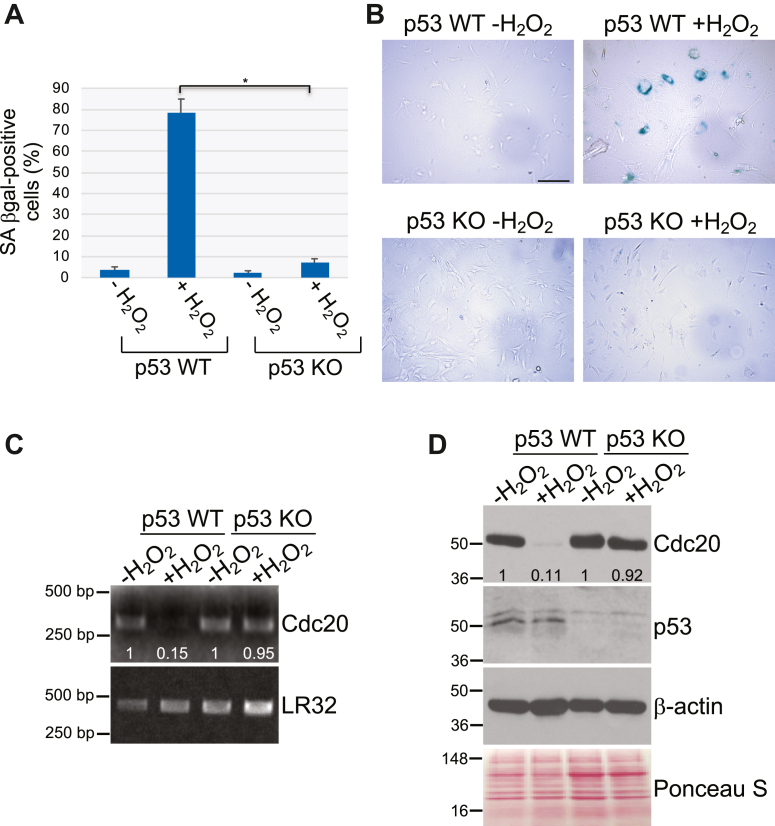
**Downregulation of Cdc20 protein and mRNA expression occur in a p53-dependent manner.***A* and *B*, mouse embryonic fibroblasts (MEFs) derived from WT and p53 KO mice were treated with sublethal hydrogen peroxide (150 μM) for 2 h to induce premature senescence. Cells were washed with PBS and recovered in complete medium for 14 days. Untreated cells were used as control. Cells were then stained to detect senescence-associated β-galactosidase activity. Quantification is shown in (*A*), representative images are shown in (*B*). The scale bar represents 100 μm. Values in (*A*) represent means ± SEM; statistical comparisons were made using the student’s *t* test. ∗*p* < 0.001. *C*, p53 WT and p53 KO MEFs were subjected to oxidative stress as described in (*A*). After 14 days, Cdc20 mRNA levels were determined by RT-PCR using primers specific for Cdc20. RT-PCR using primers specific for LR32 was performed as internal control. Quantification of mRNA band intensity is shown at the bottom of the blot. *D*, p53 WT and p53 KO MEFs were subjected to oxidative stress as described in (*A*). After 14 days, cells were collected and cell lysates were subjected to immunoblotting analysis using antibody probes specific for Cdc20 and p53. Immunoblotting with anti-β-actin IgGs was done as control. Ponceau S staining shows equal total protein loading. Quantification of protein band intensity is shown at the bottom of the blot.

The transcription factor p53 can induce cellular senescence. Interestingly, p53 has been shown to negatively regulate Cdc20 expression in human cells (50, 51). Thus, we asked whether p53 plays a role in the downregulation of Cdc20 expression that we observed in senescent cells. To directly answer this question, we induced premature senescence in p53 WT and p53 KO mouse embryonic fibroblasts (MEFs) by exposing the cells to sublethal oxidative stress. We find that oxidative stress induced premature senescence in p53 WT but not p53 KO MEFs (Fig. 3, *A* and *B*). Interestingly, sublethal oxidative stress downregulated both Cdc20 mRNA (Fig. 3*C*) and protein (Fig. 3*D*) expression in WT MEFs but not p53 KO MEFs. We conclude that p53 transcriptionally downregulates Cdc20 expression in senescent fibroblasts.

### Downregulation of Cdc20 is sufficient to induce premature senescence in human fibroblasts

**Figure 4:**
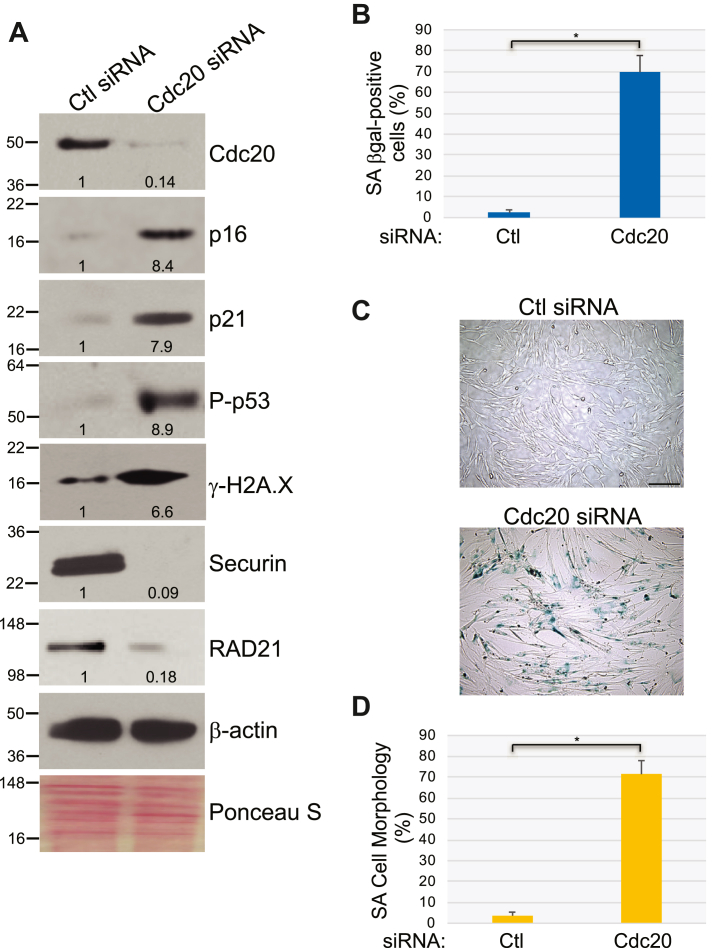
**Downregulation of Cdc20 expression induces premature senescence in WI-38 fibroblasts.***A*, human diploid WI-38 fibroblasts were transfected with either control (Ctl) or Cdc20 siRNA. Cells were cultured for 10 days. Cells were collected and cell lysates were subjected to immunoblotting analysis using antibody probes specific for Cdc20, p16, p21, phospho-p53 (P-p53), γ-H2A.X, securin, and RAD21. Immunoblotting with anti-β-actin IgGs was performed as control. Ponceau S staining shows equal total protein loading. Quantification of protein band intensity is shown at the bottom of each blot. *B*–*D*, Cdc20 protein expression was downregulated in WI-38 fibroblasts as described in (*A*). After 10 days, cells were stained to detect senescence-associated β-galactosidase (SA β-gal) activity. Quantification is shown in (*B*), representative images are shown in (*C*). The scale bar represents 100 μm. The percentage of cells possessing enlarged and flat morphology (SA cell morphology) is shown in (*D*). Values in (*B*) and (*D*) represent means ± SEM; statistical comparisons were made using the student’s *t* test. ∗*p* < 0.001.

To determine whether the downregulation of Cdc20 expression that we described in senescent human fibroblasts is correlative or causal in nature, we downregulated Cdc20 expression by siRNA in WI-38 fibroblasts that were not subjected to any extracellular stress (Fig. 4*A*). We show that downregulation of Cdc20 protein expression induced the upregulation of markers of cellular senescence such as p16, p21, phospho-p53, and γ-H2A.X after 10 days (Fig. 4*A*). We also find that most Cdc20 siRNA-expressing cells became positive for SA-β-gal activity (Fig. 4, *B* and *C*) and displayed senescence-associated (SA) cell morphology (Fig. 4*D*). Moreover, downregulation of Cdc20 by siRNA significantly inhibited cell proliferation, as shown by cell count analysis (Fig. S3*B*) and BrdU incorporation assays (Fig. S3*C*). Downregulation of Cdc20 expression for less than 10 days did not induce a maximal senescence response (not shown). Thus, downregulation of Cdc20 expression in human fibroblasts is causally linked to the development of premature senescence.

**Figure 5:**
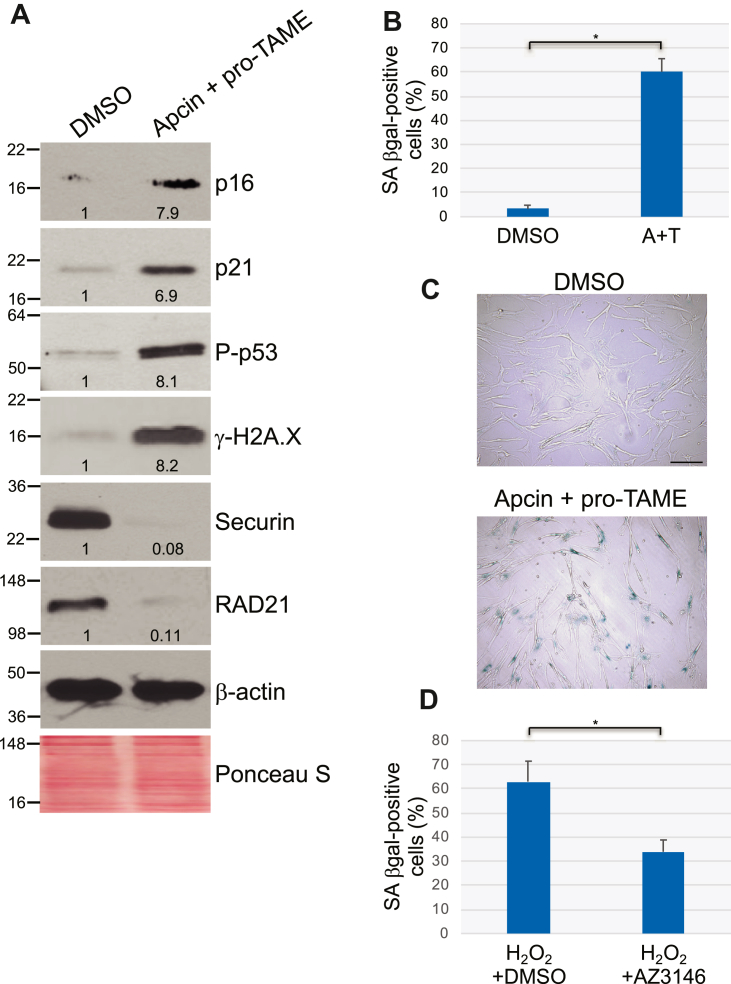
**Inhibition of APC/C promotes premature senescence in WI-38 fibroblasts.***A*, human diploid WI-38 fibroblasts were cultured for 10 days in the presence of Apcin (50 μM) and pro-TAME (40 μM). Treatment with DMSO served as control. Cells were collected and cell lysates were subjected to immunoblotting analysis using antibody probes specific for p16, p21, phospho-p53 (P-p53), γ-H2A.X, securin, and RAD21. Immunoblotting with anti-β-actin IgGs was performed as control. Ponceau S staining shows equal total protein loading. Quantification of protein band intensity is shown at the bottom of each blot. *B*–*C*, WI-38 cells were treated with Apcin (50 μM) and pro-TAME (40 μM) (A + T). After 10 days, cells were stained to detect senescence-associated β-galactosidase (SA β-gal) activity. Quantification is shown in (*B*), representative images are shown in (*C*). The scale bar represents 100 μm. *D*, WI-38 fibroblasts were treated with sublethal oxidative stress (450 μM H_2_O_2_ for 2 h) and recovered in complete medium in the presence of 1 μM AZ3146. WI-38 cells were recovered in the presence of DMSO as control. Cells were then subjected to senescence-associated β-galactosidase (SA β-gal) activity staining. Quantification of the percentage of cells that resulted positive to SA β-gal activity is shown. Values in (*B*) and (*D*) represent means ± SEM; statistical comparisons were made using the student’s *t* test. ∗*p* < 0.001. DMSO, dimethyl sulfoxide.

Cdc20 is an activator of APC/C. To independently confirm the causal relationship between loss of Cdc20 expression and development of a senescent phenotype, we tested the functional consequence of inhibiting APC/C by treating WI-38 cells with Apcin and pro-TAME, known specific inhibitors of APC/C. We find that inhibition of APC/C by Apcin plus TAME was sufficient to induce premature senescence in WI-38 fibroblasts, as shown by the upregulation of p16, p21, phospho-p53, and γ-H2A.X (Fig. 5*A*), the expression of SA-β-gal activity (Fig. 5, *B* and *C*), and the acquisition of SA cell morphology (Fig. S4*A*). Mps1 kinase is critical for APC/C inhibition and inhibition of Mps1 activates APC/C. In support of our data, treatment of WI-38 cells with the selective Mps1 inhibitor AZ3146 inhibited premature senescence induced by oxidative stress in WI-38 cells (Fig. 5*D*). Interestingly, since oxidative stress downregulated Cdc20 expression, our result of Fig. 5*D* suggests that APC/C can be activated by Mps1 inhibition even in the absence of Cdc20 and that such activation is sufficient to partially rescue SIPS. In support of this conclusion, data show that APC/C has basal activity even in the absence of Cdc20, that is, APC/C can bind substrates at low affinity in the absence of Cdc20 (52). Alternatively, one can speculate that APC/C is activated in a Cdc20-independent manner following inhibition of Mps1 by AZ3146 or that inhibition of Mps1 promotes SIPS in an APC/C-independent manner.

The Cullin subunit Apc2 is part of the catalytic core of APC/C. To determine whether oxidative stress also changed APC/C expression, we quantified the expression levels of Apc2 in WI-38 cells treated with sublethal H_2_O_2_. We find that H_2_O_2_ treatment induced a partial loss of Apc2 expression in WI-38 cells and only after 10 days (Fig. S4*B*), suggesting that oxidative stress inhibits the APC/C complex mostly by inducing the downregulation of the APC/C activator Cdc20. Together, our findings indicate that downregulation of Cdc20 protein expression is causally linked to the development of a premature senescent phenotype through inactivation of APC/C.

### Phosphorylation-dependent downregulation of securin mediates premature senescence induced by the downregulation of Cdc20

Activation of APC/C promotes the degradation of securin and triggers anaphase and mitotic exit (35, 36, 37, 38). Surprisingly, we find that securin was almost undetectable in WI-38 fibroblasts following either downregulation of Cdc20 by siRNA (Fig. 4*A*) or treatment with the APC/C inhibitors Apcin and pro-TAME (Fig. 5*A*). In support of these findings, securin expression was also virtually undetectable in senescent WI-38 cells following treatment with oxidative stress (Fig. 1*A*), UV-C light (Fig. S1*A*), or CSE (Fig. S2*A*), in which Cdc20 expression was also lost. Degradation of securin releases the protease separase, which promotes degradation of the cohesin complex allowing sister chromatids to move to opposite spindle poles. RAD21 is a structural component of the cohesion complex. Consistent with a loss of securin expression in senescent cells, RAD21 protein expression was also dramatically downregulated in senescent WI-38 cells (Fig. 1*A*, S1*A*, and S2*A*), as well as in cells induced to senescence by either the downregulation of Cdc20 (Fig. 4*A*) or inhibition of APC/C (Fig. 5*A*).

**Figure 6:**
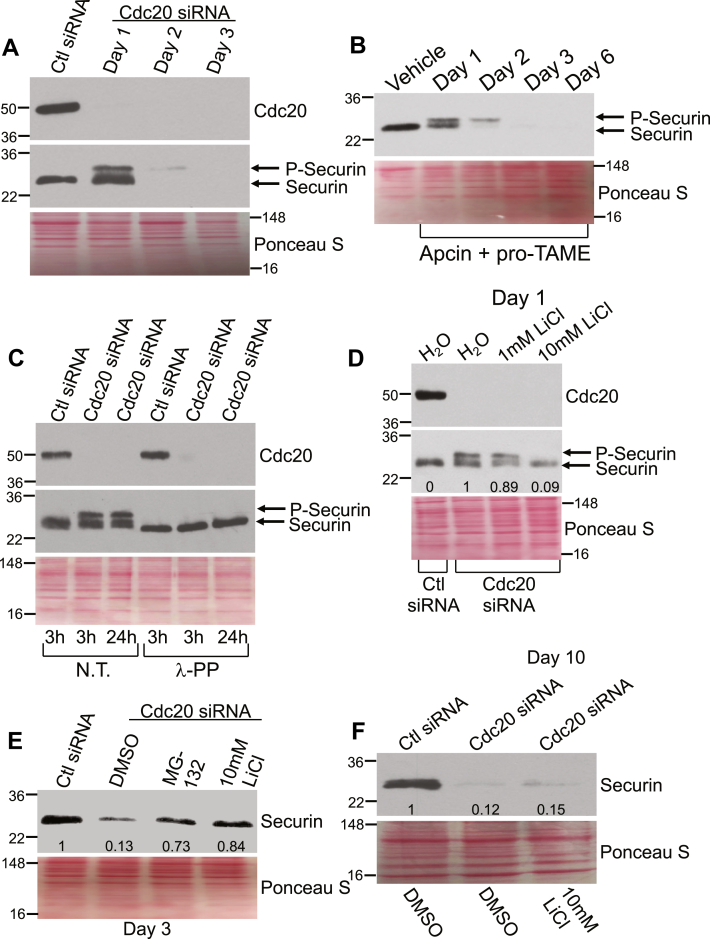
**Inhibition of the Cdc20-APC/C pathway promotes phosphorylation-dependent degradation of securin in WI-38 human fibroblasts.***A*, WI-38 cells were transfected with Cdc20 siRNA and cultured for 1, 2, and 3 days. Transfection with control (Ctl) siRNA was used as control. Cells were collected and the expression of Cdc20 and securin was determined by immunoblotting analysis using anti-Cdc20 and anti-securin IgGs. Ponceau S staining shows equal total protein loading. *B*, WI-38 fibroblasts were treated with Apcin (50 μM) and pro-TAME (40 μM) for 1, 2, 3, and 6 days. Treatment with DMSO served as control. Cells were collected and cell lysates were subjected to immunoblotting analysis using an antibody probe specific for securin. Ponceau S staining shows equal total protein loading. *C*, Cdc20 siRNA was transfected in WI-38 fibroblasts. Transfection with control (Ctl) siRNA was performed as control. After 1 day, cell lysates were incubated with lambda protein phosphatase (λ-PP; 800U) for either 3 or 24 h. Untreated samples (N.T.) served as control. Cell lysates were then subjected to immunoblotting analysis with antibody probes specific for Cdc20 and securin. Ponceau S staining shows equal total protein loading. *D*, WI-38 fibroblasts were transfected with either control (Ctl) or Cdc20 siRNA in the presence of either 1 mM or 10 mM lithium chloride (LiCl). Treatment with water (H_2_O) served as control. After 1 day, cells were collected and the expression of Cdc20 and securin was determined by immunoblotting analysis using anti-Cdc20 and anti-securin IgGs. Ponceau S staining shows equal total protein loading. Quantification of P-securin is shown at the bottom of the blot. *E*, WI-38 cells were transfected with Cdc20 siRNA. Transfection with control (Ctl) siRNA was used as control. Cells were cultured for 3 days in the presence of either MG-132 (10 μM) or LiCl (10 mM). Treatment with DMSO served as control. Cells were collected and cell lysates were subjected to immunoblotting analysis with an antibody probe specific for securin. Ponceau S staining shows equal total protein loading. Quantification of protein band intensity is shown at the bottom of the blot. *F*, WI-38 cells were transfected with Cdc20 siRNA. Transfection with control (Ctl) siRNA was used as control. Cells were cultured for 10 days in the presence of LiCl (10 mM). Treatment with DMSO served as control. Cells were collected and cell lysates were subjected to immunoblotting analysis with an antibody probe specific for securin. Ponceau S staining shows equal total protein loading. Quantification of protein band intensity is shown at the bottom of the blot. DMSO, dimethyl sulfoxide.

What is the mechanism underlying the downregulation of securin following loss of Cdc20 expression? Interestingly, we find that a higher molecular weight form of securin appeared in WI-38 cells shortly (day 1) after downregulation of Cdc20 by siRNA (Fig. 6*A*) or APC/C inhibition with Apcin and pro-TAME (Fig. 6*B*), before securin expression was lost at later time points (days 3 and 6). We demonstrate that this higher molecular weight form of securin was a phosphorylated form of the protein, as shown by the ability of λ protein phosphatase to prevent its formation in WI-38 fibroblasts 1 day after downregulation of Cdc20 by siRNA (Fig. 6*C*). Data show that glycogen synthase kinase 3-β (GSK3β) can phosphorylate securin (53, 54). Interestingly, we find that treatment with lithium chloride (LiCl), a GSK3β inhibitor, reduced the level of phosphorylated securin on day 1 (Fig. 6*D*) and rescued securin expression in Cdc20 siRNA-expressing WI-38 cells on day 3 (Fig. 6*E*). We also observed rescue of securin expression in Cdc20 siRNA-expressing WI-38 cells that were treated with the proteasome inhibitor MG-132 (Fig. 6*E*), suggesting that phosphorylated securin is degraded through the proteasome.

**Figure 7:**
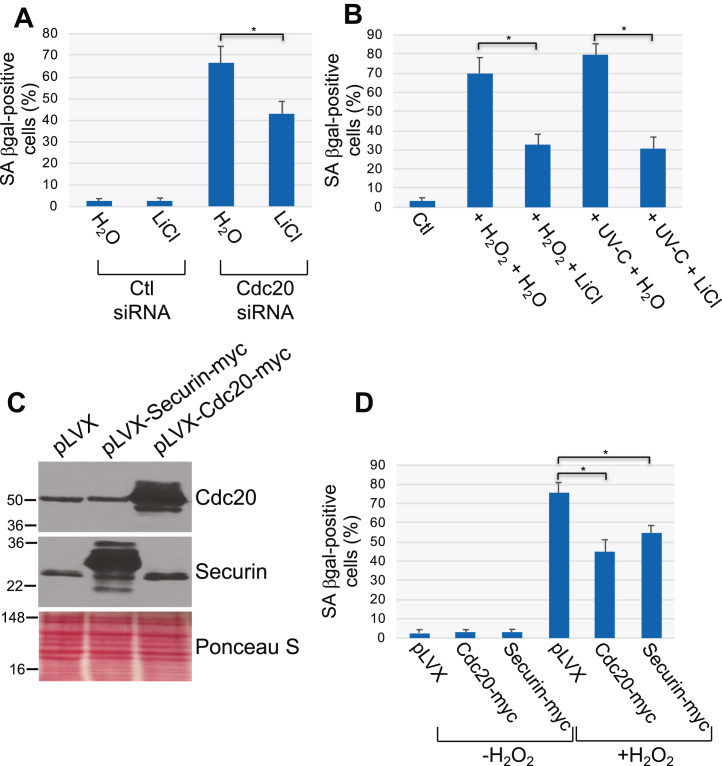
**Treatment with LiCl inhibits senescence induced by Cdc20 siRNA, H**_**2**_**O**_**2**_**, and UV-C.** Overexpression of either Cdc20 or securin inhibit oxidative stress–induced premature senescence. *A*, WI-38 fibroblasts were transfected with either control (Ctl) or Cdc20 siRNA and cultured for 10 days in the presence of 10 mM LiCl. Treatment of transfected cells with H_2_O was used as control. Cells were then subjected to senescence-associated β-galactosidase (SA β-gal) activity staining. Quantification is shown. *B*, WI-38 cells were treated with sublethal doses of either oxidative stress (450 μM H_2_O_2_ for 2 h) or UV-C (10 J/m^2^) in the presence of either 10 mM LiCl or H_2_O. Untreated cells served as control (Ctl). Quantification of cellular senescence was performed following senescence-associated β-galactosidase (SA β-gal) activity staining. (*C*, myc-tagged securin and myc-tagged Cdc20 were overexpressed in WI-38 cells by lentiviral infection using the viral vector pLVX. Overexpression of Cdc20 and securin was verified by immunoblotting analysis using antibody probes specific for securin and Cdc20. Ponceau S staining shows equal total protein loading. *D*, WI-38 fibroblasts were infected with pLVX, pLVX-securin-myc, and pLVX-Cdc20-myc. After 2 days, cells were treated to sublethal oxidative stress (450 μM H_2_O_2_ for 2 h) and recovered in complete medium for 14 days. Untreated cells served as control (-H_2_O_2_). Cells were then subjected to senescence-associated β-galactosidase (SA β-gal) activity staining. Quantification is shown. Values in (*A*), (*B*), and (*D*) represent means ± SEM; statistical comparisons were made using the student’s *t* test. ∗*p* < 0.005.

What is the functional consequence of phosphorylation-mediated securin downregulation? To directly answer this question, we assessed the ability of the GSK3β inhibitor LiCl to prevent premature senescence induced by Cdc20 siRNA in WI-38 fibroblasts. We show in Fig. 7*A* that treatment with LiCl significantly limited the percentage of senescent WI-38 cells following downregulation of Cdc20. Interestingly, LiCl treatment also inhibited SIPS caused by either oxidative stress or UV-C light (Fig. 7*B*). In support of these findings, we demonstrate that overexpression of either Cdc20 or securin (Fig. 7*C*) inhibited premature senescence induced by oxidative stress (Fig. 7*D*). Finally, we overexpressed Cdc20 in WI-38 cells and treated the cells with H_2_O_2_ and found that H_2_O_2_ treatment downregulated both Cdc20 and securin expression in vector-infected cells (Fig. S5*A*). In contrast, H_2_O_2_ did not downregulate Cdc20 expression and only partially downregulated securin expression in Cdc20 overexpressing cells (Fig. S5*A*). Consistent with these data, the partial inhibition of H_2_O_2_-induced senescence by the overexpression of Cdc20 was abolished by securin siRNA (Fig. S5*B*). Together, our data show that downregulation of Cdc20 inactivates APC/C, leading to premature cellular senescence through a phosphorylation-dependent degradation of securin.

Importantly, we have confirmed our data on the functional relationship between Cdc20 expression and induction of premature senescence using normal human bronchial epithelial (NHBE) cells. More specifically, we find that Cdc20 protein (Fig. S6*A*) and mRNA (Fig. S6*B*) expression was downregulated in senescent NHBE cells following stimulation with sublethal oxidative stress. In addition, we find that downregulation of Cdc20 in NHBE cells, using siRNA, was sufficient to induce premature senescence, as shown by upregulation of the senescence markers p16, p21 (Fig. S6*C*), and SA-β-galactosidase activity (Fig. S6*D*). Finally, we find that downregulation of Cdc20 in NHBE cells induced securin phosphorylation followed by securin downregulation (Fig. S6*E*).

### Cdc20 expression is upregulated in lung adenocarcinoma and downregulation of Cdc20 in NSCLC cells promotes apoptosis

**Figure 8:**
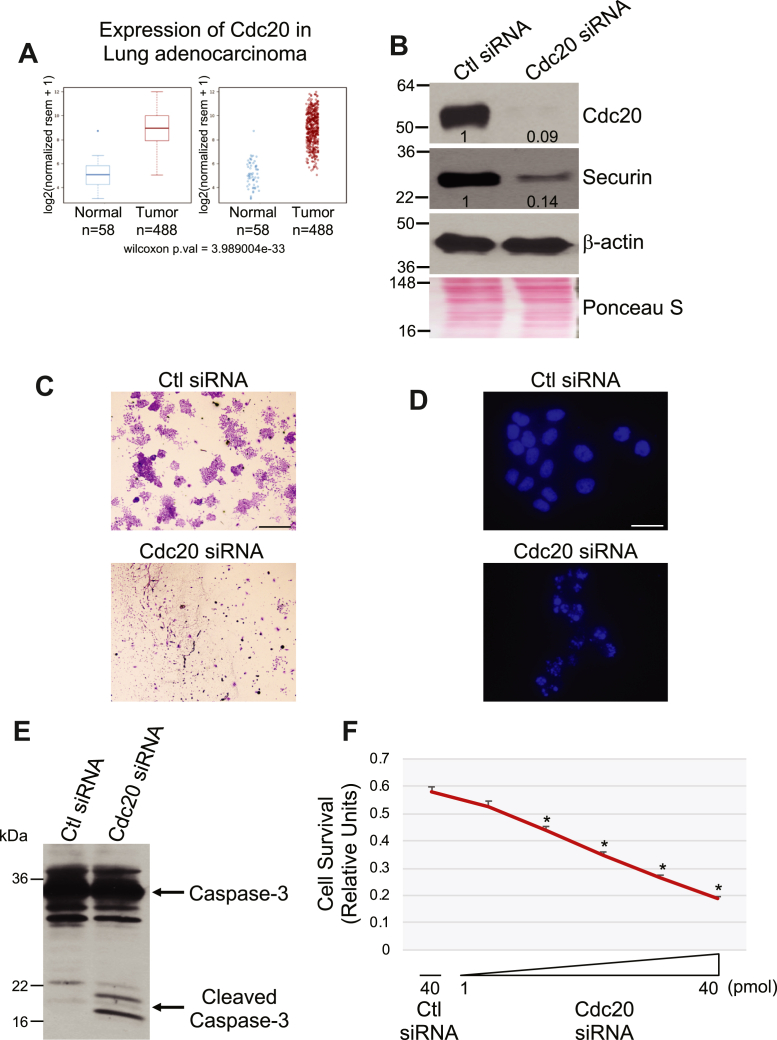
**Downregulation of Cdc20 induces apoptosis in H460 cells.***A*, gene expression analysis of Cdc20 in normal lung and in the lung of adenocarcinoma patients using The Cancer Genome Atlas database. *B*, H460 cells were transfected with either control (Ctl) or Cdc20 siRNA. After 48 h, cells were collected and cell lysates were subjected to immunoblotting analysis with antibody probes specific for Cdc20 and securin. Immunoblotting with anti-β-actin IgGs was performed as control. Ponceau S staining shows equal total protein loading. Quantification of protein band intensity is shown at the bottom of each blot. *C*–*E*, H460 cells were transfected with either control (Ctl) or Cdc20 siRNA. After 48 h, cells were subjected to crystal violet staining (*C*) (Scale bar = 2 mm), DAPI staining (*D*) (Scale bar = 50 μm), and immunoblotting analysis with anti-caspase-3 IgGs (*E*). *F*, H460 cells were transfected with increasing concentrations of Cdc20 siRNA (2.5, 5, 10, 20, and 40 pmol). As control, H460 cells were transfected with 40 pmol of control (Ctl) siRNA. After 48 h, cell survival was quantified by MTT assay. Quantification is shown. Values in (*F*) represent means ± SEM; statistical comparisons were made using the student’s *t* test. ∗*p* < 0.001.

Aberrant expression of Cdc20 and securin has been described in a number of cancers, including lung adenocarcinoma. TCGA database analysis did indeed show increased expression of both Cdc20 (Fig. 8*A*) and securin (Fig. S7*A*) in lung adenocarcinoma, as compared to control tissue. We then asked whether downregulation of Cdc20 in NSCLC cells promotes senescence like we described in normal human fibroblasts. To answer this question, we downregulated Cdc20 expression in H460 NSCLC (Fig. 8*B*). Interestingly, we did not observe the development of a senescence phenotype in H460 cells carrying very low levels of Cdc20 (data not shown). In contrast, we noticed that most cells started to die within 24 h. In fact, cell number (Fig. S7*B*), BrdU incorporation (Fig. S7*C*), and positive crystal violet staining (Fig. 8*C*) were drastically reduced in Cdc20 siRNA-carrying H460 cells, as compared with H460 transfected with control siRNA. We also find that reduced Cdc20 expression induced nuclear condensation (Fig. 8*D* and S7*D*), upregulated the expression of cleaved caspase-3 (Fig. 8*E*), and inhibited cell survival (Fig. 8*F*), as shown by 4,6-diamidino-2-phenylindole (DAPI) staining, immunoblotting analysis, and 3-(4,5-dimethylthiazol-2-yl)-2,5-diphenyltetrazolium bromide (MTT) assay, respectively. Consistent with increased apoptosis in H460 cells with low Cdc20 expression, Cdc20 siRNA-expressing cells formed fewer colonies in soft agar assays (Fig. S7, *E* and *F*). Virtually identical results were obtained with A549 cells, another NSCLC line (data not shown).

**Figure 9:**
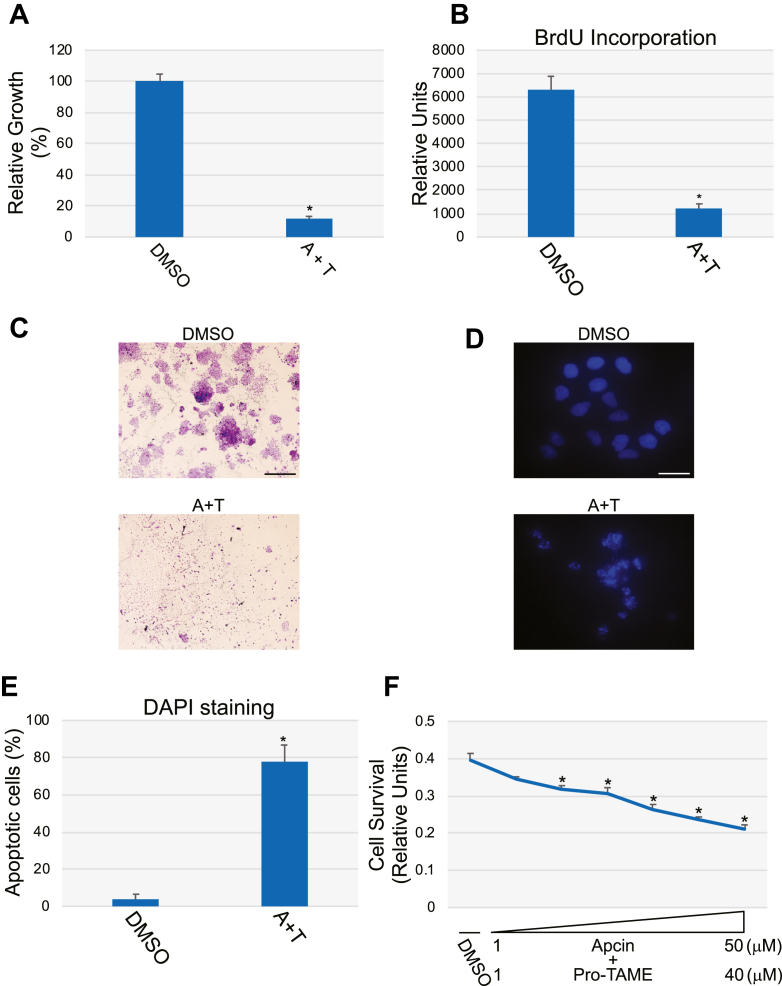
**Inhibition of the Cdc20-APC/C pathway promotes apoptosis in H460 cells.***A*–*E*, H460 cells were treated with Apcin (50 μM) and pro-TAME (40 μM) (A + T) for 48 h. Treatment with DMSO served as control. Attached cells were then counted with a hemocytometer (*A*), subjected to bromodeoxyuridine (BrdU) incorporation assays (*B*), and crystal violet staining (*C*) (Scale bar = 2 mm). Cells were also stained with DAPI (Scale bar = 50 μm). Representative images are shown in (*D*), quantification of cells with fragmented nuclei is shown in (*E*). *F*, H460 cells were treated with increasing concentrations of Apcin (1.56, 3.125, 6.25, 12.5, 25, and 50 μM) and pro-TAME (1.25, 2.5, 5, 10, 20, and 40 μM) for 48 h. Cell survival was then quantified by MTT assay. Values in (*A*), (*B*), (*E*), and (*F*) represent means ± SEM; statistical comparisons were made using the student’s *t* test. ∗*p* < 0.001.

Consistent with the data obtained following downregulation of Cdc20, we find that inhibition of APC/C with Apcin and pro-TAME also resulted in cell death of H460 cells. In fact, Apcin/TAME-treated H460 cells displayed significantly reduced proliferation (Fig. 9*A*), BrdU incorporation (Fig. 9*B*), and crystal violet staining (Fig. 9*C*), as well as increased nuclear condensation (Fig. 9, *D* and *E*) and reduced cell survival (Fig. 9*F*), as compared with H460 cells treated with vehicle. Very similar results were obtained with A549 cells (data not shown). We conclude from these data that downregulation of Cdc20 is sufficient to induce apoptotic cell death in NSCLC through inhibition of APC/C activity.

### Chemotherapeutic drugs downregulate Cdc20 expression and either downregulation of Cdc20 or inhibition of APC/C makes NSCLC cells more sensitive to chemotherapeutic drug-induced apoptosis

**Figure 10:**
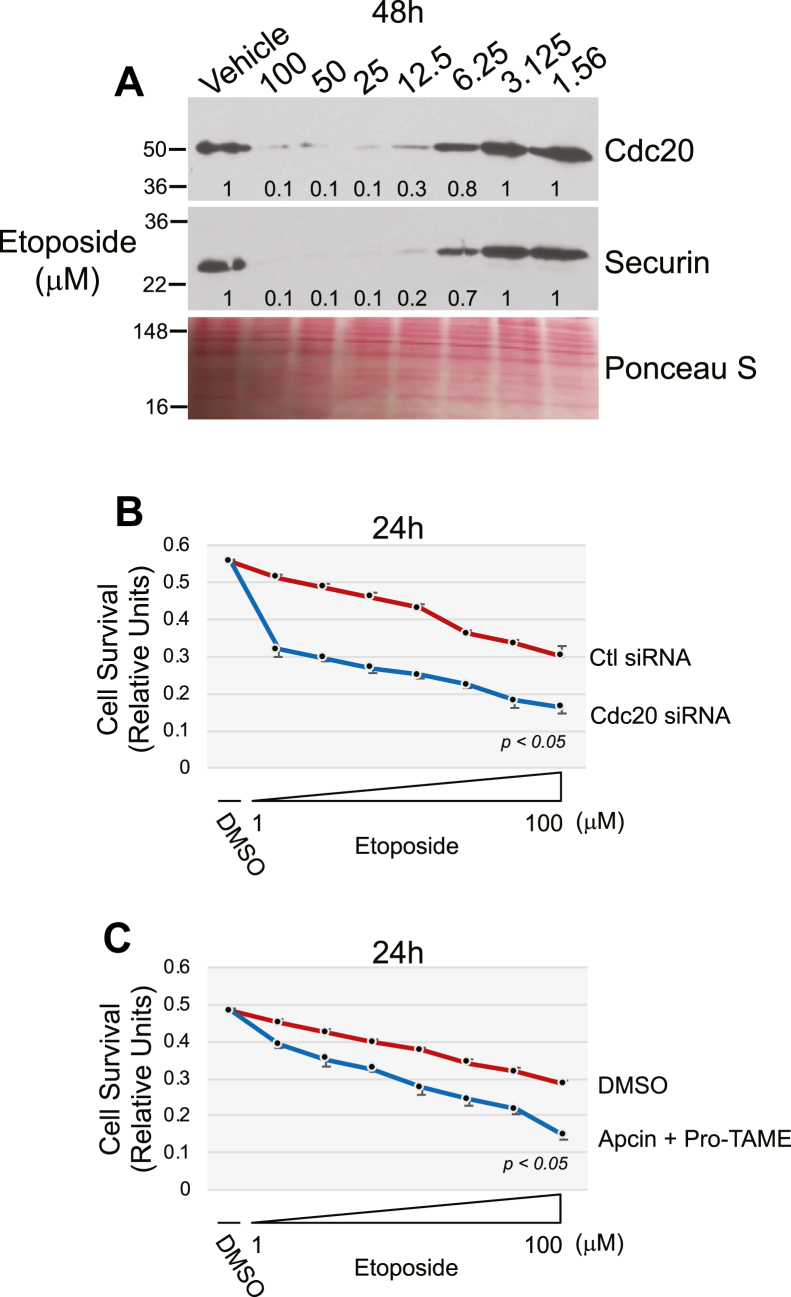
**Etoposide downregulates Cdc20 and securin expression in H460 cells.** Inhibition of the Cdc20-APC/C pathway sensitizes H460 cells to etoposide-induced cell death. *A*, H460 cells were treated with increasing concentrations of etoposide (1.56 μM, 3.125 μM, 6.25 μM, 12.5 μM, 25 μM, 50 μM, and 100 μM) for 48 h. Cells were then collected and cell lysates were subjected to immunoblotting analysis using antibody probes specific for Cdc20 and securin. Ponceau S staining shows equal total protein loading. Quantification of protein band intensity is shown at the bottom of each blot. *B*, H460 cells were transfected with either control (Ctl) or Cdc20 siRNA (10 pmol) and cultured in the presence of either DMSO or different concentrations of etoposide (1.56 μM, 3.125 μM, 6.25 μM, 12.5 μM, 25 μM, 50 μM, and 100 μM) for 24 h. Cell survival was quantified by MTT assay. *C*, H460 cells were treated with Apcin (50 μM) and pro-TAME (40 μM) in the presence of either DMSO or different concentrations of etoposide (1.56 μM, 3.125 μM, 6.25 μM, 12.5 μM, 25 μM, 50 μM, and 100 μM) for 24 h. Cell survival was quantified by MTT assay. Values in (*B*) and (*C*) represent means ± SEM; statistical comparisons were made using the student’s *t* test. ∗*p* < 0.001. DMSO, dimethyl sulfoxide.

One of the main modes of action of chemotherapeutic drugs is the activation of apoptosis in cancer cells. Since our data show that either downregulation of Cdc20 or inhibition of APC/C induced apoptotic cell death, we asked whether chemotherapeutic drugs induce Cdc20 downregulation. To this end, we treated H460 NSCLC cells with increasing concentrations of etoposide (from 1.56 μM to 100 μM) and assessed Cdc20 protein expression by immunoblotting analysis. We find that increasing concentrations of etoposide resulted in a proportional downregulation of Cdc20 protein expression (Fig. 10*A*). We also find that etoposide downregulated Cdc20 mRNA expression (Fig. S8). Similarly, cisplatin (Fig S9*A*) and 5-fluoracil (5-FU) (Fig. S10*A*) downregulated Cdc20 expression in H460 cells to almost undetectable levels. Interestingly, downregulation of Cdc20 by siRNA made H460 more sensitive to etoposide-induced cell death (Fig. 10*B*). Similarly, inhibition of APC/C enhanced H460 cell death induced by etoposide (Fig. 10*C*). Etoposide treatment did not change the expression of APC/C, as shown by the inability of etoposide to alter Apc2 protein expression (Fig. S11). Sensitization of chemotherapeutic drug–induced apoptosis by downregulation of Cdc20 and inhibition of APC/C was not limited to etoposide. In fact, we show that H460 cells either transfected with Cdc20 siRNA or treated with Apcin plus TAME were more sensitive to apoptosis induced by either cisplatin (Fig. S9, *B* and *C*) or 5-FU (Fig. S10, *B* and *C*). Thus, loss of Cdc20/APC/C pathway activation makes H460 NSCLC cells more sensitive to chemotherapeutic drug–induced apoptotic cell death.

We also find that cell death induced by deregulation of the Cdc20/APC/C pathway is not limited to NSCLC cells. We show in Fig. S12*A* that either downregulation of Cdc20 by siRNA or inactivation of APC/C with Apcin/TAME treatment drastically reduced cell survival of HCT-116 colorectal cancer cells as well as MCF-7 and MDA-MB-231 breast cancer cells. Interestingly, p53 seems to act as a negative regulator of Cdc20 expression both under resting conditions and following chemotherapeutic drug treatment in cancer cells. In fact, Cdc20 expression was significantly lower in untreated HCT-116 p53 WT cells, as compared to isogenic HCT-116 p53 null cells (Fig. S12*B*). In addition, while etoposide downregulated Cdc20 expression (as well as securin expression) (Fig. S12*C*) and induced cell death (Fig. S12*D*) in HCT-116 p53 WT cells, it failed to do so in HCT-116 p53 null cells (Fig. S12, *C* and *D*).

### Downregulation of Cdc20 expression promotes phosphorylation-dependent downregulation of securin. Inhibition of securin phosphorylation inhibits apoptosis induced by either downregulation of Cdc20 or chemotherapeutic drugs

**Figure 11:**
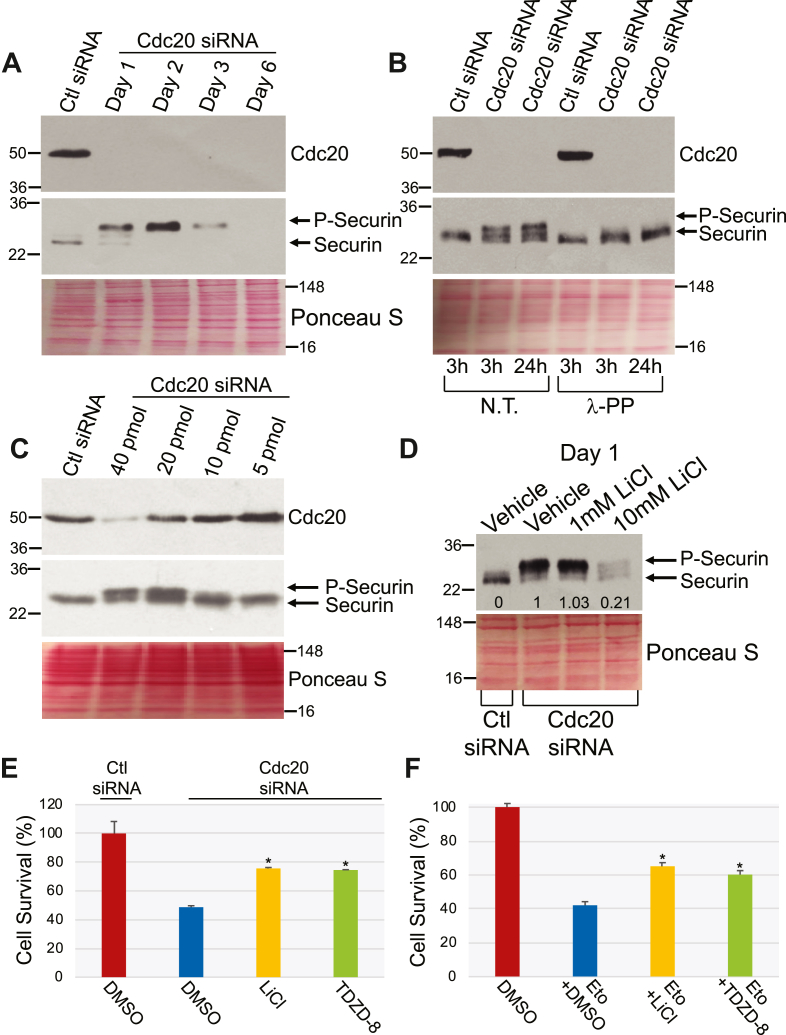
**Downregulation of Cdc20 promotes phosphorylation-dependent loss of securin in H460 cells.** LiCl and TDZD-8 inhibit cell death induced by either Cdc20 siRNA or etoposide. *A*, H460 cells were transfected with Cdc20 siRNA and cultured for 1, 2, 3, and 6 days. Transfection with control (Ctl) siRNA served as control. Cells were then collected and cell lysates subjected to immunoblotting analysis with anti-Cdc20 and anti-securin IgGs. Ponceau S staining shows equal total protein loading. *B*, Cdc20 siRNA was transfected in H460 cells. Transfection with control (Ctl) siRNA was performed as control. After 1 day, cell lysates were incubated with lambda protein phosphatase (λ-PP; 800U) for either 3 or 24 h. Untreated samples (N.T.) served as control. Cell lysates were then subjected to immunoblotting analysis with antibody probes specific for Cdc20 and securin. Ponceau S staining shows equal total protein loading. *C*, different amounts of Cdc20 siRNA (5, 10, 20, and 40 pmol) were transfected in H460 cells. Control (Ctl) siRNA was transfected as control. After 1 day, cells were collected and expression of Cdc20 and securin was determined by immunoblotting analysis with anti-Cdc20 and anti-securin IgGs. Ponceau S staining shows equal total protein loading. *D*, H460 cells were transfected with Cdc20 siRNA and cultured for 24 h in the presence of LiCl (1 mM and 10 mM). Transfection with control (Ctl) siRNA and treatment with H_2_O served as controls. Cells were then collected and securin expression was detected by immunoblotting analysis with anti-securin IgGs. Ponceau S staining shows equal total protein loading. Quantification of P-securin is shown at the bottom of the blot. *E*, H460 cells were transfected with Cdc20 siRNA and treated with either LiCl (10 mM) or TDZD-8 (1 μM) for 48 h. Transfection with control (Ctl) siRNA and treatment with DMSO served as controls. Cell survival was then quantified by MTT assays. *F*, H460 cells were treated with etoposide (100 μM) in the presence of either LiCl (10 mM) or TDZD-8 (1 μM) for 48 h. Treatment with DMSO served as control. Cell survival was then quantified by MTT assays. Values in (*E*) and (*F*) represent means ± SEM; statistical comparisons were made using the student’s *t* test. ∗*p* < 0.005. DMSO, dimethyl sulfoxide.

Since we find loss of securin expression in normal human fibroblasts after downregulation of Cdc20 expression, we asked whether the same was true in H460 cells. We find that downregulation of Cdc20 by siRNA induced a secondary loss of securin expression in H460 cells (Fig. 8*B*). Consistent with this result, treatment with etoposide (Fig. 10*A*), cisplatin (Fig. S9*A*), and 5-FU (Fig. S10*A*), which downregulated Cdc20 expression, also downregulated securin expression in H460 cells. We then assessed whether a phosphorylated form of securin was expressed in H460 cells, following downregulation of Cdc20, before total securin expression was lost in the absence of Cdc20. Similar to what we observed in WI-38 fibroblasts, we show in Fig. 11*A* that downregulation of Cdc20 initially induced the formation of a higher molecular weight band of securin followed by the complete loss of securin expression. We then demonstrate that this higher molecular weight form of securin represents phosphorylated securin, as shown by its disappearance when Cdc20 siRNA-transfected H460 lysates were treated with λ protein phosphatase (Fig. 11*B*). Phosphorylation of securin does not require the total loss of Cdc20 since we detected phosphorylated securin even when Cdc20 was only partially downregulated by ∼50% with 20 pmol of Cdc20 siRNA (Fig. 11*C*).

In support of a key role of GSK3β in the phosphorylation of securin following loss of Cdc20, treatment with the GSK3β inhibitor LiCl prevented phosphorylation of securin in H460 cells transfected with Cdc20 siRNA (Fig. 11*D*). In addition, we find that downregulation of GSK3β inhibited the phosphorylation of securin induced by Cdc20 downregulation (Fig. S13*A*), and the inhibition of GSK3β with LY2090314 (a selective GSK3β inhibitor) reduced the phosphorylation of securin induced by Cdc20 downregulation (Fig. S13*B*). Consistent with these data, both downregulation of Cdc20 by siRNA and inhibition of APC/C with Apcin/pro-TAME downregulated WT securin but not a mutant form of securin in which the putative GSK3β phosphorylation sites S183 and S184 were mutated to alanines in order to disrupt the GSK3β consensus sequence SSILST (S/TXXXS/T) at residues 183 to 188 (Fig. S14). Cyclin dependent kinase 1 (CDK1) has been reported to phosphorylate securin (55). We ruled out in Fig. S13*A* and S13*B* that CDK1 is the kinase mediating securin phosphorylation. We conclude that GSK3β controls phosphorylation of securin in the absence of an active Cdc20/APC complex.

**Figure 12:**
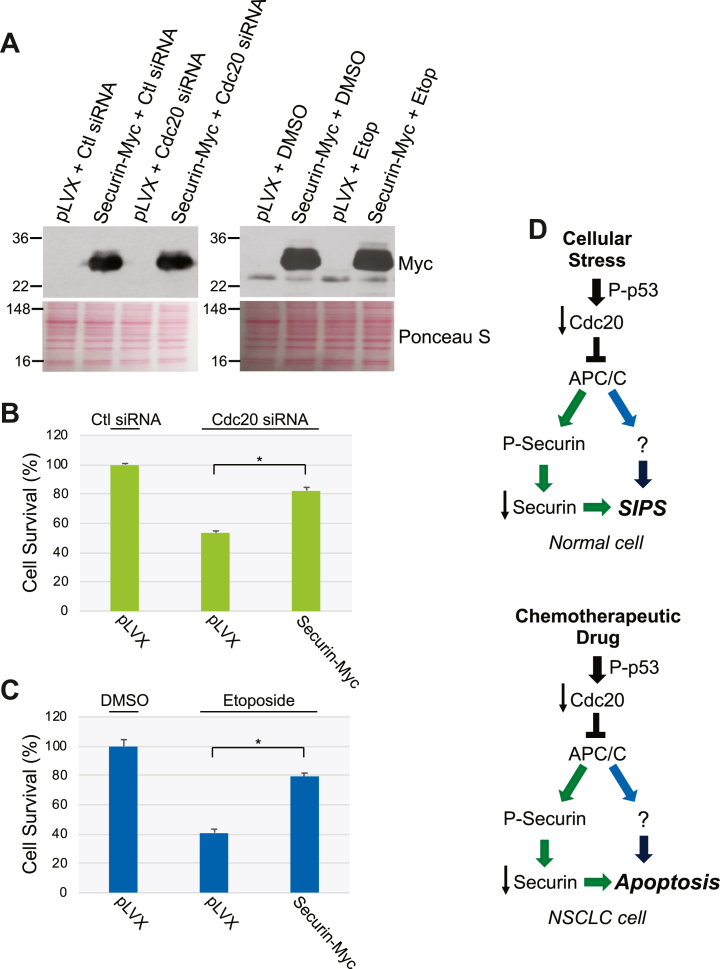
**Overexpression of securin in H460 cells inhibits cell death induced by either downregulation of Cdc20 or etoposide.***A*, H460 cells were infected with a lentiviral vector (pLVX) carrying myc-tagged securin. Infection with pLVX alone served as control. After 48 h, cells were either transfected with Cdc20 siRNA or treated with etoposide (100 μM). Transfection with control (Ctl) siRNA and treatment with DMSO served as controls. After an additional 48 h, cells were collected and expression of securin was determined by immunoblotting analysis using an antibody probe specific for myc. Ponceau S staining shows equal total protein loading. *B*, H460 cells were infected with a lentiviral vector (pLVX) carrying myc-tagged securin. Infection with pLVX alone served as control. After 48 h, cells were transfected with either control (Ctl) siRNA or Cdc20 siRNA. After an additional 48 h, cell survival was quantified by MTT assays. *C*, H460 cells were infected with a lentiviral vector (pLVX) carrying myc-tagged securin. Infection with pLVX alone served as control. After 48 h, cells were treated with etoposide (100 μM). Treatment with DMSO served as control. After an additional 48 h, cell survival was quantified by MTT assays. Values in (*B*) and (*C*) represent means ± SEM; statistical comparisons were made using the student’s *t* test. ∗*p* < 0.001. *D*, schematic diagram summarizing loss of Cdc20-dependent senescence and apoptosis. In normal cells, extracellular stress promotes the p53-dependent downregulation of Cdc20. Loss of Cdc20 promotes inhibition of the APC/C complex, leading to the phosphorylation-dependent downregulation of securin. Downregulation of securin, in conjunction with a yet unknown signaling mediator, which is downstream of loss of Cdc20/inhibition of APC/C in a securin-independent manner, promotes premature senescence. In NSCLC cells, chemotherapeutic drugs promote the p53-dependent downregulation of Cdc20, leading to inhibition of APC/C. Loss of Cdc20/inhibition of APC/C promotes the phosphorylation-dependent downregulation of securin. Downregulation of securin, in conjunction with a yet unknown signaling mediator, which is downstream of loss of Cdc20/inhibition of APC/C in a securin-independent manner, promotes apoptosis. DMSO, dimethyl sulfoxide ; NSCLC, non–small cell lung cancer.

Can LiCl restrain cell death induced by either Cdc20 downregulation or chemotherapeutic drug treatment? To answer this question, we quantified cell survival, by MTT assay, of H460 NSCLC cells transfected with Cdc20 siRNA in the presence or absence of LiCl. We find that LiCl inhibited cells death induced by the loss of Cdc20 expression (Fig. 11*E*). Consistent with this result, LiCl inhibited etoposide-induced cell death (Fig. 11*F*). These data were independently confirmed by showing that TDZD-8, a selective GSK3 inhibitor, inhibited both Cdc20 siRNA- induced and etoposide-induced cell death in H460 cells (Fig. 11, *E* and *F*). Finally, we demonstrate that overexpression of securin (Fig. 12*A*) partially rescued cell death induced by either Cdc20 siRNA (Fig. 12*B*) or etoposide (Fig. 12*C*) in H460 cells.

## Discussion

Activation of APC/C by Cdc20 is key for proper segregation of sister chromatids to the two poles of the dividing cell, ensuing anaphase and mitotic exit. Mitotic exit can be delayed in cells in which Cdc20-APC/C function is compromised, which results in a DDR signaling that is initiated during mitosis and persists and is enhanced following mitotic exit. Interestingly, recent findings show that APC/C can also be activated in response to DNA damage. Data show that APC/C activation promotes proper DNA repair (56, 57). Although the precise molecular mechanism(s) remains unknown, APC/C activation associated with DNA damage appears to occur in a p53-dependent manner (58). Thus, impaired APC/C signaling sustains a chronic DDR by inhibiting both sister chromatids segregation and DNA lesion repair. When the DNA is damaged beyond repair capacity, the cell avoids propagating potentially tumorigenic alterations by undergoing either senescence or apoptotic cell death. Our data provide evidence of a novel molecular mechanism that functionally links genotoxic stress to both APC/C function and development of cellular senescence. We find that external stimuli, such as oxidative stress, UV-C light, and CSEs, transcriptionally downregulated the expression of Cdc20, an APC/C activator, in a p53-dependent manner, and induced senescence in normal human diploid fibroblasts. We also provide evidence that inhibition of APC/C is causally linked to the development of a DDR and the acquisition of a senescent phenotype, as shown by the ability of either Cdc20 siRNA or APC/C inhibitors to activate p53, upregulate the double-strand break marker γ-H2A.X, and induce senescence in human fibroblasts in the absence of external stimuli. We also find that either downregulation of Cdc20 or inhibition of APC/C downregulated the expression of the double-strand break repair protein RAD21. Together, our data show that inhibition of Cdc20-APC/C signaling causes the development of cellular senescence in normal human fibroblasts through a mechanism that is associated with a chronic DDR.

Activation of APC/C leads to the degradation of securin, which triggers metaphase–anaphase transition (35, 36, 37, 38). Thus, one would not expect downregulation of securin following inhibition of APC/C by either Cdc20 downregulation or Apcin/TAME treatment. Unexpectedly, we observed a drastic downregulation of securin in human fibroblasts in which the Cdc20-APC/C signaling was inhibited. Similarly, securin expression was almost lost in cells treated with senescence-inducing stimuli, which also downregulated Cdc20 expression. Downregulation of securin occurred, at least in part, through the proteasome pathway and in a manner that was dependent of its phosphorylation. Interestingly, while the downregulation of Cdc20 by siRNA was sufficient to induce premature senescence, downregulation of securin by siRNA failed to both inhibit cell proliferation and promote the expression of markers of cellular senescence (data not shown). Considering that the overexpression of either Cdc20 or securin inhibited SIPS, we conclude that downregulation of Cdc20 is necessary and sufficient to promote senescence while downregulation of securin is necessary but not sufficient.

Intriguingly, treatment with the GSK-3β inhibitor, lithium chloride, inhibited securin phosphorylation, securin degradation, and SIPS. Thus, it is possible that both the initial phosphorylation of securin and its subsequent downregulation promote senescence following loss of Cdc20-APC/C function (Fig. 12*D*). This scenario would be consistent with data showing that both the presence of securin and its further degradation are necessary to arrest cell proliferation in response to ionizing radiations (59). Alternatively, loss of Cdc20 may activate a yet unknown and securin-independent mechanism that, in combination with loss of securin, is necessary to induce SIPS (Fig. 12*D*). Moreover, we find that LiCl rescued securin expression 3 days (Fig. 6*E*) but not 10 days (Fig. 6*F*) after Cdc20 downregulation. Considering that treatment with LiCl inhibited premature senescence induced by either Cdc20 siRNA (Fig. 7*A*) or H_2_O_2_ and UV-C (Fig. 7*B*) and that overexpression of securin partially inhibited senescence induced by H_2_O_2_ (Fig. 7*D*), these data suggest that the early loss of securin expression, rather than securin loss at later time point, after downregulation of Cdc20 by either siRNA or H_2_O_2_/UV-C treatment, plays a major role in the signaling that leads to premature senescence. We conclude that loss of securin promotes the onset rather than the maintenance of cellular senescence. Given the known importance of cellular senescence in aging and age-related diseases, we propose the Cdc20-APC/C-securin pathway as a signaling of novel relevance to the aging field that deserves further investigation. In support of this conclusion, a germline missense mutation of c.856C>A (p.R286S) in Cdc20 was found in a patient with premature aging syndrome showing random chromosome number instabilities (60).

Evasion from cellular senescence is necessary in order for lung preneoplasias to progress to lung cancer. In fact, senescence has been observed in preneoplastic lesions of the lung but not in lung adenocarcinomas (24, 25, 26, 27, 28, 29, 30). The signaling pathways that contribute to senescence evasion remain to be fully established. Since our data demonstrate that loss of Cdc20/securin–mediated signaling promotes senescence, one would anticipate elevated expression of these two proteins in lung cancer cells. In fact, both Cdc20 and securin are overexpressed in NSCLC. We speculate that increased expression of both proteins is one of the mechanisms through which lung cancer cells, and possibly other cancer cell types, evade senescence to drive tumorigenesis. Can we induce senescence of NSCLC cells by downregulating Cdc20 or securin expression or by inhibiting APC/C? Our data show that either downregulation of Cdc20 and securin or inhibition of APC/C is not sufficient to promote senescence in NSCLC cells, in contrast to what we observed in normal human lung fibroblasts. Inhibition of the Cdc20-APC/C signaling resulted instead in apoptotic cell death in both H460 and A549 NSCLC cells. Consistent with a key role that inhibition of the Cdc20-APC/C-securin pathway plays in apoptosis of NSCLC cells, chemotherapeutic drugs, which promoted apoptosis of H460 cells, downregulated both Cdc20 and securin expression.

While the functional outcome of inhibiting the Cdc20-APC/C signaling was different in NSCLC cells and normal lung fibroblasts (*i.e*., apoptosis and senescence, respectively), we observed a number of similarities between the two cell types. First, either downregulation of Cdc20 by siRNA or inhibition of APC/C induced a phosphorylation-dependent downregulation of securin in both WI-38 and H460 cells. Second, downregulation of Cdc20 and securin occurred in a p53-dependent manner in both WI-38 cells following stimulation with senescence-inducing stimuli and H460 cells following treatment with chemotherapeutic drugs. Third, inhibition of GSK-3β inhibited both senescence of WI-38 cells following Cdc20 downregulation or stimulation with senescence-inducing stimuli and apoptosis of H460 cells induced by Cdc20 siRNA or etoposide treatment. Fourth, similarly to what we observed in WI-38 cells with respect to senescence, downregulation of securin is not sufficient to promote apoptosis in H460 (Fig. S15, *A*–*C*). Finally, overexpression of either Cdc20 or securin inhibited the functional outcomes initiated by the loss of Cdc20/APC/C signaling in both WI-38 and H460 cells (*i.e*., senescence and apoptosis, respectively). Thus, it appears that the same p53-dependent loss of Cdc20-APC/C-securin signaling that promotes senescence in normal cells, drives a cellular response that favors apoptotic cell death upon cell transformation (Fig. 12*D*). Interestingly, either downregulation of Cdc20 or inhibition of APC/C sensitizes NSCLC cells to chemotherapeutic drug–induced apoptosis. Thus, genetic and/or pharmacologic interventions aimed at inhibiting Cdc20-APC/C-securin signaling may be a valid strategy to either enhance cancer cell death induced by chemotherapeutic drugs or limit undesired side effects caused by chemotherapy in NSCLC. In addition, inhibition of Cdc20-APC/C-securin signaling, through a mechanism that does not rely on the p53-dependent downregulation of Cdc20, may be a valid approach to induce/enhance cell death in NSCLC cells carrying mutant p53, in which chemotherapeutic drugs fail to downregulate Cdc20 in the absence of WT p53.

## Experimental procedures

### Materials

Antibodies were obtained from the following sources: anti-Cdc20 (14866), anti-securin (13445), anti-phospho-p53 (9284), anti-γ-H2A.X (9718), Apc2 (12301), and anti-Rad21 (4321) IgGs were from Cell Signaling Technologies; anti-β-actin (mAb C4), anti-p21 (F-5), anti-p53 (6243); anti-c-*myc* (mAb 9E10) IgGs were from Santa Cruz Biotechnology; anti-p16 ARC (mAb EP1551Y) was from Abcam; anti-Caspase-3 (ADI-AAP-113) IgG was from Enzo Life Sciences, Inc. Apcin, pro-TAME, and TDZD-8 were from Cayman Chemical. 5-FU, cisplatin, etoposide, AZ3146, lithium chloride, and chloroquine were from Sigma–Aldrich. Lamba protein phosphatase was from New England Biolabs. MG-132 was from EMD Millipore. Silencer Select negative control siRNA, Silencer Select Cdc20 siRNA, and Silencer Select Securin siRNAs were from Thermo Fisher Scientific. All other biochemical reagents were of the highest available purity and were commercially obtained.

### Cell culture

MEFs, MDA-MB-231, and MCF-7 cells were cultured in Dulbecco's modified Eagle's medium (DMEM) supplemented with 2 mM glutamine, 100 U/ml penicillin, 100 μg/ml streptomycin, and 10% fetal bovine serum. WI-38 human lung fibroblasts were cultured in Eagle’s minimal essential medium supplemented with 2 mM glutamine, 100 U/ml penicillin, 100 μg/ml streptomycin, and 10% fetal bovine serum. NHBE cells were obtained from Lonza and cultured in SAGM BulletKit media. A549 and H460 cells were cultured in Ham’s F12 medium, supplemented with 2 mM glutamine, 100 U/ml penicillin, 100 μg/ml streptomycin, and 10% fetal bovine serum. HCT-116 cells were cultured in McCoy 5 alpha medium.

### Induction of premature senescence

#### SIPS

H_2_O_2_-induced senescence. Cells were treated with sublethal doses of H_2_O_2_ (450 μM) for 2 h. Cells were washed twice with PBS and cultured in complete medium for 14 days.

UV-C-induced senescence. Cells were irradiated with a sublethal dose of UV-C light (10/m^2^). During irradiation, cells were deprived of growth medium. Cells were allowed to recover in complete medium for 14 days.

CSE-induced senescence. CSE solutions were prepared as described previously (61). Through one opening of a stopcock, 10 ml of sterile DMEM were drawn into a 50 ml plastic syringe. Subsequently, 40 ml of cigarette smoke were drawn into the syringe and mixed with the medium by vigorous shaking. One cigarette was used for each 10 ml of medium. The generated CSE solution was filtered (0.22 μm) to remove large particles. The resulting solution was designated a 100% CSE solution. The CSE solution was used immediately after generation. Cells were cultured with 2% CSE for 14 days.

We have chosen to quantify premature senescence 14 days after stimulation with the senescence-inducing stimuli described previously because we and others have shown that only the chronic stimulation with these stimuli promotes the development of a senescent phenotype (61, 62, 63, 64, 65).

#### Replicative senescence

We have induced replicative senescence in WI-38 fibroblasts by culturing the cells until about 50 cumulative population doublings. After 50 cumulative population doublings, WI-38 cells loose proliferative capacity and enter replicative senescence (66, 67). Replicative senescence in Fig. 2, *D* and *E* was verified by a lack of BrdU incorporation and the acquisition of senescence-associated β-galactosidase activity in at least 90% of cells (not shown).

### Immunoblotting

Cells were collected in boiling sample buffer. Cellular proteins were resolved by SDS-PAGE (12.5% acrylamide; 15% for detection of phosphorylated securin) and transferred to Amersham Protran 0.2 μm nitrocellulose blotting membrane (GE Healthcare Life Sciences). Blots were incubated for 1 h 15 min in TBST (10 mM Tris–HCl, pH 8.0, 150 mM NaCl, 0.2% Tween 20) containing 2% powdered skim milk and 1% bovine serum albumin. After three washes with TBST, membranes were incubated overnight with the primary antibody and washed three more times with TBST. Blots were then incubated for 1 h 15 min with horseradish peroxidase–conjugated goat anti-rabbit/mouse IgG. Bound proteins were detected using an ECL detection kit (Pierce) according to the manufacturer’s protocol. Representative images are shown. Quantification of protein expression was done: sample protein concentration was determined using the bicinchoninic acid assay kit from Sigma–Aldrich. Equal total proteins were resolved by SDS-PAGE. Equal protein loading was assessed by Ponceau S staining. Bands were scanned, and their density was quantified and normalized based on Ponceau S intensity.

### Senescence-associated β-galactosidase activity assay

Senescence-associated β-galactosidase activity was measured using the Senescence β-Galactosidase Staining Kit according to the manufacturer’s protocol (Cell Signaling Technology). Average percent senescence was calculated from quantification of total cells and senescent cells in 10 fields of view per condition, using an upright epifluorescent Leica microscope. Representative images of microscope fields are shown.

### Senescence-associated cell morphology

It is well known that senescent cells can be easily distinguished from proliferating cells due to their typical large and flat morphology (1, 6, 64, 68). Cells were examined using a BX50WI Optical light microscope (Olympus) at a magnification of 10. The percentage of cells showing senescence-like (flat and large) cell morphology in 10 fields of view was recorded per condition.

### Transfection of siRNA

siRNA was introduced into cells using Lipofectamine RNAiMax reagent from Life Technologies according to the manufacturer’s protocol, using 40 pmol per well of 6-well culture plates.

### RNA isolation and RT-PCR

Cells were collected and total RNA was isolated using the RNeasy Mini kit from Qiagen. Equal amounts of RNA were treated with RNase-free DNase and subjected to reverse transcription using the Advantage RT-for-PCR kit from Clontech, according to the manufacturer’s protocol. PCR was then performed for each gene studied in its linear zone of amplification using the following primers: Cdc20 forward-ggcaccagtgatcgacacatt; Cdc20 reverse-gggtccaactcaaaacagcgc; GAPDH forward-gcaaattccatggcaccgt; GAPDH reverse-tcgccccacttgattttgg.

### BrdU incorporation assay

Cell proliferation was measured and quantified using the Cell Proliferation ELISA, BrdU (colorimetric) kit (Roche). The kit was used according to the manufacturer’s protocol. Cells were incubated with BrdU labeling solution overnight at 37 °C. The absorbances were measured at 370 nm following substrate incubation using an ELISA plate reader.

### Growth in soft agar

Cells (5 × 10^4^) were suspended in 3 ml of complete medium and 0.33% SeaPlaque low-melting temperature agarose. These cells were plated over a 2 ml layer of solidified complete medium and 0.5% agarose and allowed to settle to the interface between these layers at 37 °C. After 30 min, the plates were allowed to harden at room temperature (RT) for 30 min before returning to 37 °C. After 10 days, colonies were photographed under low magnification. The colonies in 60 randomly chosen fields from three independent plates were counted.

### Lentivirus preparation

Myc-tagged human Cdc20 and myc-tagged human securin were cloned into the lentiviral vector pLVX using routine cloning techniques. Lentivirus was generated by cotransfecting lentiviral vectors with pMD2G and pSpAX2 into 293T cells using the calcium phosphate method. Forty-eight hours after transfection, cell culture medium was collected and viral supernatant was filtered using a 0.45 nm filter. A myc-tagged mutant form of securin was generated in which the putative GSK3β phosphorylation sites S183 and S184 were mutated to alanines in order to disrupt the GSK3β consensus sequence SSILST (S/TXXXS/T) at residues 183 to 188. This mutant form of securin was then cloned into the lentiviral vector pLVX using standard cloning techniques.

### Crystal violet staining

Cells were stained with crystal violet by incubating the cells with 10% crystal violet in 70% ethanol for 2 min followed by extensive washes with PBS. Quantification of crystal violet staining from three independent experiments was performed using Image J software analysis (National Institutes of Health).

### DAPI staining

Cells (untreated or treated with chemotherapeutic drugs) were harvested and incubated with staining buffer (PBS containing 3.7% paraformaldehyde, 0.1% Triton, 10 μg/ml RNase A, and 1 μg/ml DAPI) at RT for 1 h. Nuclear morphology was examined under an Olympus Provis fluorescent microscope. A total of 1200 cells were scored from four independent viewing areas from three independent experiments for each experimental point.

### MTT assay

The MTT assay kit (M6494) was purchased from Thermo Fisher Scientific and assay was performed according to the manufacturer’s recommendations.

## Statistical analysis

Studies were performed in triplicates using three biological replicates to achieve statistically significant differences. The average ± SEM) is shown. Significance was calculated using the Student’s *t* test.

## Data availability

All of the data are contained within the article.

## Supporting information

This article contains supporting information.

## Conflict of interest

The authors declare that they have no conflicts of interest with the contents of this article.
