# Supplementary material for: Impaired Cdc20 signaling promotes senescence in normal cells and apoptosis in non–small cell lung cancer cells
Source: J Biol Chem. 2022 Aug 19;298(10):102405. doi: 10.1016/j.jbc.2022.102405 (PMC9490043; doi:10.1016/j.jbc.2022.102405)

SUPPLEMENTARY FIGURE LEGENDS

Figure S1. Sub-lethal doses of UV-C downregulate both Cdc20 and securin expression and induce premature senescence in WI-38 fibroblasts. *(A)* Human diploid WI-38 fibroblasts were treated with sub-lethal doses of UV-C (10 J/m^2). Cells were washed with PBS and recovered in complete medium for 14 days. Cells were collected and cell lysates were subjected to immunoblot analysis using antibody probes specific for phospho-p53 (P-p53), γ-H2A.X, Cdc20, securin, and RAD21. Untreated cells (Ctl) were used as control. Ponceau S staining shows equal total protein loading. Quantification of protein band intensity is shown at the bottom of each blot. *(B-D)* Premature senescence was induced in WI-38 cells as described in (A). Cells were then stained to detect senescence-associated β-galactosidase activity. Representative images are shown in (B), quantification is shown in (C). Scale bar = 100 μm. The percentage of cells possessing enlarged and flat morphology [senescence-associated (SA) cell morphology] is shown in (D). Values in C and D represent means ± SEM; statistical comparisons were made using the student's t-test. *P<0.001.

Figure S2. Cigarette smoke extracts downregulate both Cdc20 and securin expression and induce premature senescence in WI-38 fibroblasts. (A) Human diploid WI-38 fibroblasts were treated with sub-lethal doses of cigarette smoke extracts (2%) for 14 days. Cells were collected and cell lysates were subjected to immunoblot analysis using antibody probes specific for phospho-p53 (P-p53), γ-H2A.X, Cdc20, securin, and RAD21. Untreated cells (Ctl) were used as control. Ponceau S staining shows equal total protein loading. Quantification of protein band intensity is shown at the bottom of each blot. *(B-D)* Premature senescence was induced in WI-38 cells as described in (A). Cells were then stained to detect senescence-associated β-galactosidase activity. Representative images are shown in (B), quantification is shown in (C). Scale bar = 100 μm. The percentage of cells possessing enlarged and flat morphology [senescence-associated (SA) cell morphology] is shown in (D). Values in C and D represent means ± SEM; statistical comparisons were made using the student's t-test. *P<0.001.

Figure S3. Proteasome inhibition does not rescue oxidative stress-induced downregulation of Cdc20 in WI-38 cells. Downregulation of Cdc20 by siRNA inhibits WI-38 cell proliferation. *(A)* WI-38 human diploid fibroblasts were treated with sub-lethal levels of oxidative stress (450μM for 2 hours). Cells were then washed with PBS and recovered in complete medium for 14 days in the presence of the proteasome inhibitors MG132 (10μM), Chloroquine (50 μM), or ammonium chloride (NH_4Cl; 10 mM). Unstressed cells and cells treated with DMSO served as controls. Cells were then collected and cell lysates were subjected to immunoblotting analysis using anti-Cdc20 and anti-β-actin IgGs. Ponceau S staining shows equal total protein loading. Quantification of protein band intensity is shown at the bottom of the blot. *(B,C)* WI-38 cells were transfected with either control (Ctl) or Cdc20 siRNA and cultured for 10 days. In (A), cells were counted using a hemocytometer. In (B), cell proliferation was quantified by BrdU incorporation assay. Values in B and C represent means ± SEM; statistical comparisons were made using the student's t-test. *P<0.001.

Figure S4. Inhibition of APC/C in WI-38 fibroblasts promotes the acquisition of senescence-associated cell morphology. Oxidative stress only marginally downregulates Apc2. (A) WI-38 human diploid fibroblasts were treated with apcin (50μM) and pro-TAME (40μM) (A+T) for 10 days. Treatment with DMSO served as control. The percentage of cells possessing enlarged and flat morphology [senescence-associated (SA) cell morphology] is shown. (B) WI-38 cells were treated with 450 μM H_2O_2 for 2 hours and recovered in complete medium for 1, 3, 6 and 10 days. Cells were then collected and cell lysates were subjected to immunoblotting analysis using anti-Apc2 IgGs. Ponceau S staining shows equal total protein loading. Quantification of protein band intensity is shown at the bottom of the blot. Values in (A) represent means ± SEM; statistical comparisons were made using the student's t-test. *P<0.001.

Figure S5. Over-expression of Cdc20 partially rescues oxidative stress-induced downregulation of securin. Downregulation of securin prevents the inhibition of SIPS induced by the over-expression of Cdc20. *(A)* Myc-tagged Cdc20 was over-expressed in WI-38 cells by lentiviral infection using the viral vector pLVX. After 2 days, cells were treated to sub-lethal oxidative stress (450μM H_2O_2 for 2 hours) and recovered in complete medium for 14 days. Infection with the empty pLVX vector and untreated cells were used as controls. Cells were collected and cell lysates were subjected to immunoblotting analysis using antibody probes specific for Cdc20 and securin. Ponceau S staining shows equal total protein loading. Quantification of protein band intensity is shown at the bottom of each blot. *(B)* Myc-tagged Cdc20 was over-expressed in WI-38 cells by lentiviral infection using the viral vector pLVX. After 1 day, cells were transfected with either control (Ctl) or securin siRNA. After 1 day, cells were treated to sub-lethal oxidative stress (450μM H_2O_2 for 2 hours) and recovered in complete medium for 14 days. Infection with the empty pLVX vector and untreated cells were used as controls. Cells were then subjected to senescence-associated β-galactosidase (SA β-gal) activity staining. Quantification is shown. Values in *(B)* represent means ± SEM; statistical comparisons were made using the student's t-test. **P*<0.005.

Figure S6. Oxidative stress downregulates Cdc20 protein and mRNA expression in NHBE cells. Downregulation of Cdc20 expression induces premature senescence in NHBE cells. *(A,B)* Normal human bronchial epithelial (NHBE) cells were treated with sub-lethal hydrogen peroxide (450μM) for 2 hours. Cells were washed with PBS and recovered in complete medium for 14 days. Untreated cells were used as control. In *(A)*, cells were collected and cell lysates were subjected to immunoblot analysis using an antibody probe specific for Cdc20. Ponceau S staining shows equal total protein loading. Quantification of protein band intensity is shown at the bottom of the blot. In (*B*), cells were collected and the expression level of Cdc20 mRNA was determined by RT-PCR using primers specific for Cdc20. Expression of GAPDH was detected as internal control. Quantification of mRNA band intensity is shown at the bottom of the blot. *(C,D)* NHBE cells were transfected with either control (Ctl) or Cdc20 siRNA. Cells were cultured for 10 days. In *(C)*, cells were collected and cell lysates were subjected to immunoblotting analysis using antibody probes specific for Cdc20, p16, p21, and securin. Ponceau S staining shows equal total protein loading. Quantification of protein band intensity is shown at the bottom of each blot. In *(D)*, cells were stained to detect senescence-associated β-galactosidase (SA β-gal) activity. Quantification is shown. *(E)* NHBE cells were transfected with either control (Ctl) or Cdc20 siRNA. Cells were cultured for 1, 2 and 3 days. Cells were collected and cell lysates were subjected to immunoblotting analysis using antibody probes specific for Cdc20 and securin. Ponceau S staining shows equal total protein loading. Values in D represent means ± SEM; statistical comparisons were made using the student's t-test. **P*<0.001.

Figure S7. Downregulation of Cdc20 promotes apoptosis in H460 cells. *(A)* Gene expression analysis of securin in normal lung and in the lung of adenocarcinoma patients using The Cancer Genome Atlas database. *(B-D)* H460 cells were transfected with Cdc20 siRNA. Transfection with control (Ctl) siRNA served as control. After 48 hours, attached cells were counted with a hemocytometer (B), and subjected to bromodeoxyuridine (BrdU) incorporation assays (C). Cells were also stained with DAPI. Quantification of cells with fragmented nuclei is shown in (D). *(E-F)* H460 cells were transfected with Cdc20 siRNA. Transfection with control (Ctl) siRNA served as control. After 48 hours, $5x10^4$ cells were plated in agarose and cultured for 10 days. Quantification of the number of foci per field is shown in (E), representative images are shown in (F). Scale bar = 2 mm. Values in B-E represent means ± SEM; statistical comparisons were made using the student's t-test. **P*<0.001.

Figure S8. Etoposide downregulates Cdc20 mRNA expression in H460 cells. H460 cells were treated with etoposide (100 μM) for 48 hours. Treatment with DMSO served as control. Cells were collected and the expression level of Cdc20 mRNA was determined by RT-PCR using primers specific for Cdc20. Expression of GAPDH was detected as internal control. Quantification of mRNA band intensity is shown at the bottom of the blot.

Figure S9. Cisplatin downregulates Cdc20 and securin expression in H460 cells. Inhibition of the Cdc20-APC/C pathway sensitizes H460 cells to cisplatin-induced cell death. *(A)* H460 cells were treated with increasing concentrations of cisplatin (1.56 μM, 3.125 μM, 6.25 μM, 12.5 μM, 25 μM, 50 μM, 100 μM) for 48 hours. Cells were then collected and cell lysates were subjected to immunoblotting analysis using antibody probes specific for Cdc20 and securin. Ponceau S staining shows equal total protein loading. Quantification of protein band intensity is shown at the bottom of each blot. *(B)* H460 cells were transfected with either control (Ctl) or Cdc20 siRNA (10 pmol) and cultured in the presence of either DMSO or different concentrations of cisplatin (1.56 μM, 3.125 μM, 6.25 μM, 12.5 μM, 25 μM, 50 μM, 100 μM) for 24 hours. Cell survival was quantified by MTT assay. (C) H460 cells were treated with Apcin (50 μM) and pro-TAME (40 μM) in the presence of either DMSO or different concentrations of cisplatin (1.56 μM, 3.125 μM, 6.25 μM, 12.5 μM, 25 μM, 50 μM, 100 μM) for 24 hours. Cell survival was quantified by MTT assay. Values in B and C represent means ± SEM; statistical comparisons were made using the student's t-test. *P<0.05.

Figure S10. 5-FU downregulates Cdc20 and securin expression in H460 cells. Inhibition of the Cdc20-APC/C pathway sensitizes H460 cells to 5-FU-induced cell death. *(A)* H460 cells were treated with increasing concentrations of 5-FU (1.56 μM, 3.125 μM, 6.25 μM, 12.5 μM, 25 μM, 50 μM, 100 μM) for 48 hours. Cells were then collected and cell lysates were subjected to immunoblotting analysis using antibody probes specific for Cdc20 and securin. Ponceau S staining shows equal total protein loading. *(B)* H460 cells were transfected with either control (Ctl) or Cdc20 siRNA (10 pmol) and cultured in the presence of either DMSO or different concentrations of 5-FU (1.56 μM, 3.125 μM, 6.25 μM, 12.5 μM, 25 μM, 50 μM, 100 μM) for 24 hours. Cell survival was quantified by MTT assay. *(C)* H460 cells were treated with Apcin (50 μM) and pro-TAME (40 μM) in the presence of either DMSO or different concentrations of 5-FU (1.56 μM, 3.125 μM, 6.25 μM, 12.5 μM, 25 μM, 50 μM, 100 μM) for 24 hours. Cell survival was quantified by MTT assay. Values in B and C represent means ± SEM; statistical comparisons were made using the student's t-test. *P<0.05.

Figure S11. Etoposide does not alter Apc2 expression in H460 cells. H460 cells were treated with etoposide (100 μM) for 48 hours. Treatment with DMSO served as control. Cells were collected and the expression level of Apc2 protein was determined by immunoblotting analysis using anti-Apc2 IgGs. Ponceau S staining shows equal total protein loading. Quantification of protein band intensity is shown at the bottom of the blot.

Figure S12. Inhibition of the Cdc20-APC/C pathway induces cell death in breast and colon cancer cells. Downregulation of Cdc20 and securin expression by etoposide occurs in a p53-dependent manner. *(A)* Cdc20 siRNA was transfected in MCF-7 and MDA-MB-231 breast cancer cells as well as in HCT-116 colon cancer cells. Transfection with control (Ctl) siRNA was used as control. Cells were also treated with either Apcin (50 μM) and pro-TAME (40 μM) (A+T) or DMSO as control. After 48 hours, cell survival was quantified by MTT assay. *(B)* Expression of Cdc20 in p53 wild type (p53 WT) and p53 knock out (p53 KO) HCT-116 cells was determined by immunoblotting analysis using anti-Cdc20 IgGs. Ponceau S staining shows equal total protein loading. Quantification of protein band intensity is shown at the bottom of the blot. *(C,D)* p53 WT and p53 KO HCT-116 cells were treated with etoposide (100μM) for 48 hours. Treatment with DMSO served as control. In (C), cells were then collected and cell lysates subjected to immunoblotting analysis using antibody probes specific for Cdc20 and securin. Ponceau S staining shows equal total protein loading. Quantification of protein band intensity is shown at the bottom of each blot. In (D), cell survival was quantified by MTT assay. Values in A and D represent means ± SEM; statistical comparisons were made using the student's t-test. *P<0.001.

Figure S13. Downregulation of GSK3β, but not CDK1, prevents phosphorylation of securin induced by Cdc20 siRNA. *(A)* H460 cells were transfected with either control (Ctl), CDK1 or GSK3β siRNA. After 1 day, cells were transfected with either Ctl or Cdc20 siRNA. After 1 day, cells were collected and cell lysates were subjected to immunoblotting analysis using an antibody probe specific for securin. Ponceau S staining shows equal total protein loading. Quantification of P-securin is shown at the bottom of the blot. (B) H460 cells were transfected with either control (Ctl) or Cdc20 siRNA. Cells were cultured for 1 day in the presence of different concentrations of LY2090314 (10μM, 50μM, 250μM). Treatment with DMSO served as control. Cells were collected and cell lysates were subjected to immunoblotting analysis using anti-securin IgGs. Ponceau S staining shows equal total protein loading. Quantification of P-securin is shown at the bottom of the blot.

Figure S14. Cdc20 siRNA and apcin+TAME fail to downregulate a mutant form of securin lacking the GSK3β consensus sequence. H460 cells were infected with a lentiviral vector (pLVX) carrying either myc-tagged wild type securin (WT-securin-myc) or a myc-tagged mutant form of securin in which the putative GSK3β phosphorylation sites S183 and S184 were mutated to alanines in order to disrupt the GSK3β consensus sequence SSILST (S/TXXXS/T) at residues 183-188 (MUT-securin-myc). H460 cells were then either transfected with Cdc20 siRNA or treated with Apcin (50μM) and pro-TAME (40μM). Transfection with control (Ctl) siRNA and treatment with DMSO served as controls. After 48 hours, cells were collected and the expression levels of WT-securin-myc and MUT-securin-myc were determined by immunoblotting analysis with anti-c-myc IgGs. Quantification of protein band intensity is shown at the bottom of each blot.

Figure S15. Downregulation of securin does not induce cell death in H460 cells and does not sensitize H460 cells to etoposide-induced cell death. *(A,B)* H460 cells were transfected with securin siRNA and cultured for 48 hours. Transfection with control (Ctl) siRNA served as control. In (A), cells were collected and the expression of securin and Cdc20 was determined by immunoblotting analysis using specific antibody probes. Immunoblotting with anti-β-actin IgGs served as internal control. Ponceau S staining shows equal total protein loading. Quantification of protein band intensity is shown at the bottom of each blot. In (B), cell survival was quantified by MTT assay. *(C)* H460 cells were transfected with either control (Ctl) or securin siRNA. Cells were then cultured for 24 hours in the presence of either different concentrations of etoposide (1.56 μM to 100μM) or DMSO. Cell survival was quantified by MTT assay. Values in B and C represent means ± SEM; statistical comparisons were made using the student's t-test. NS: not statistically significant.

Volonte et al.,
Figure S1

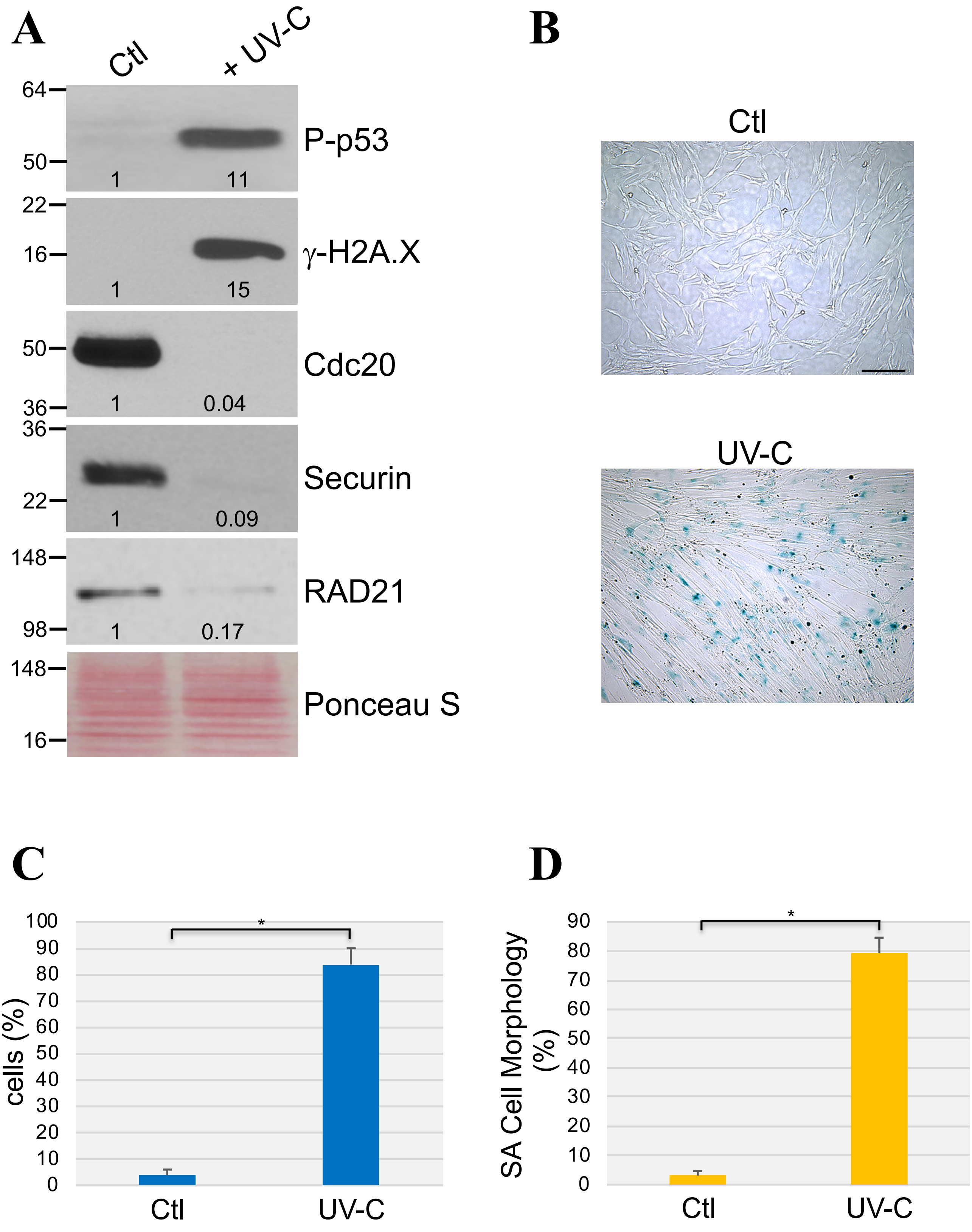

Volonte et al.,
Figure S2

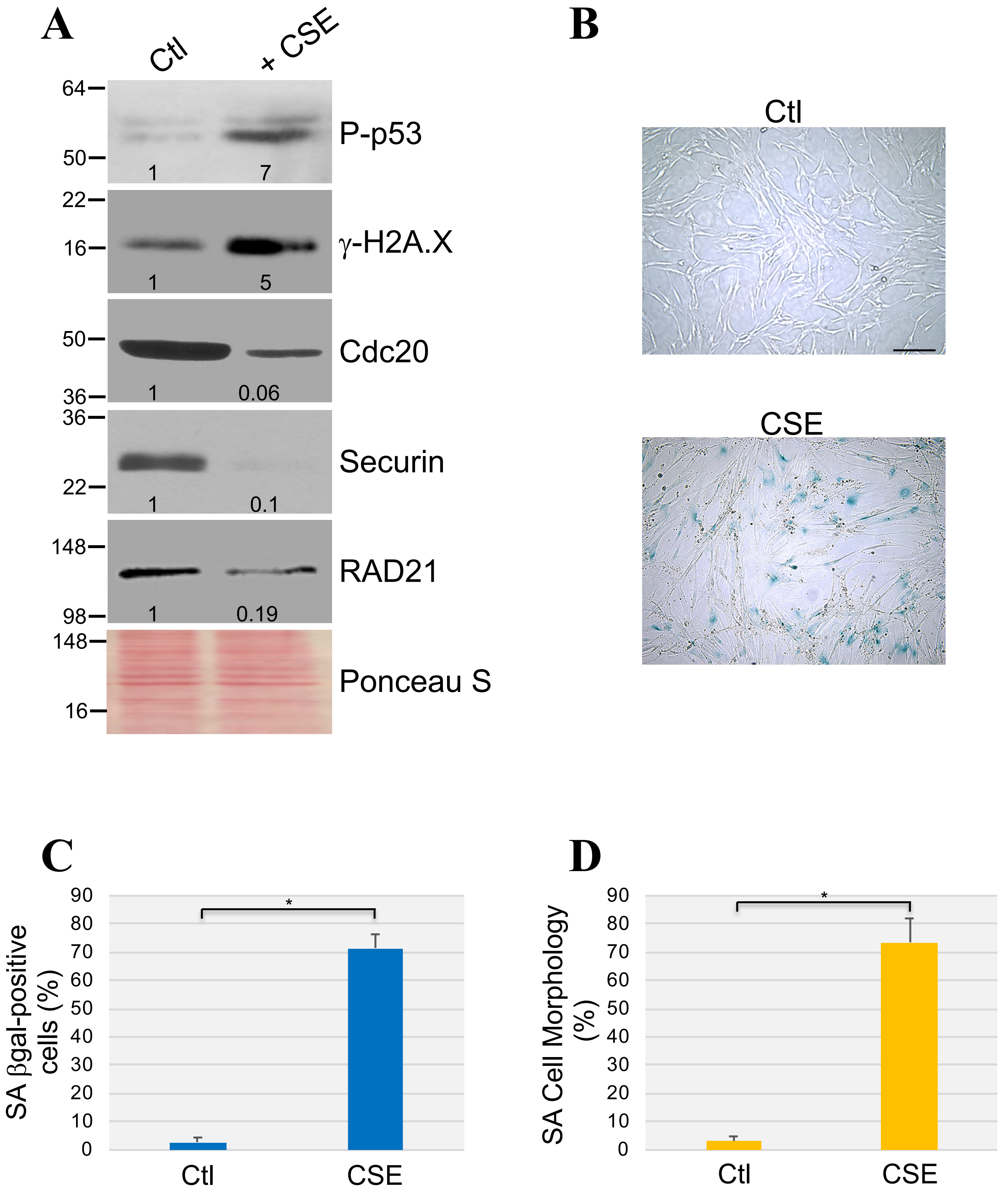

Volonte et al.,
Figure S3

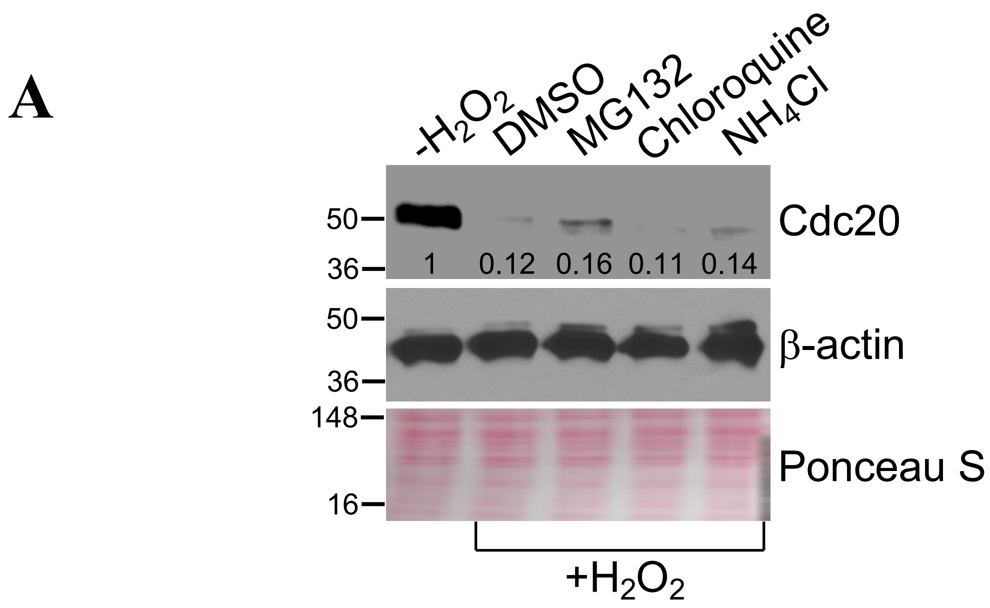

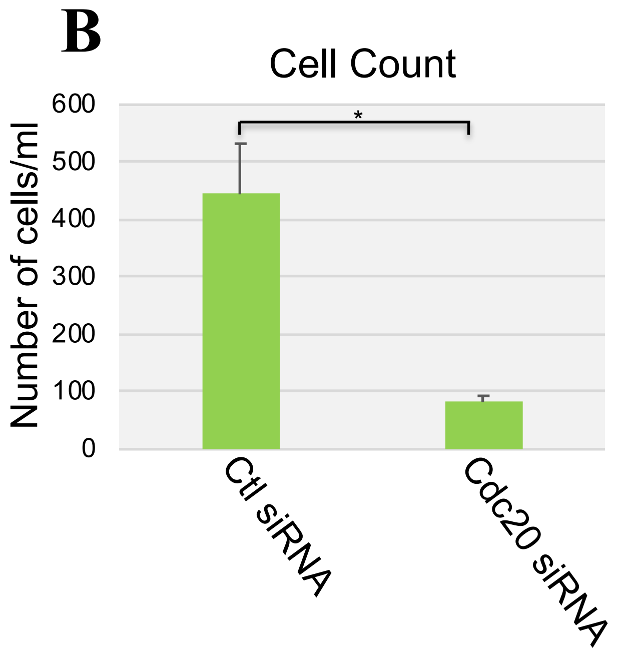

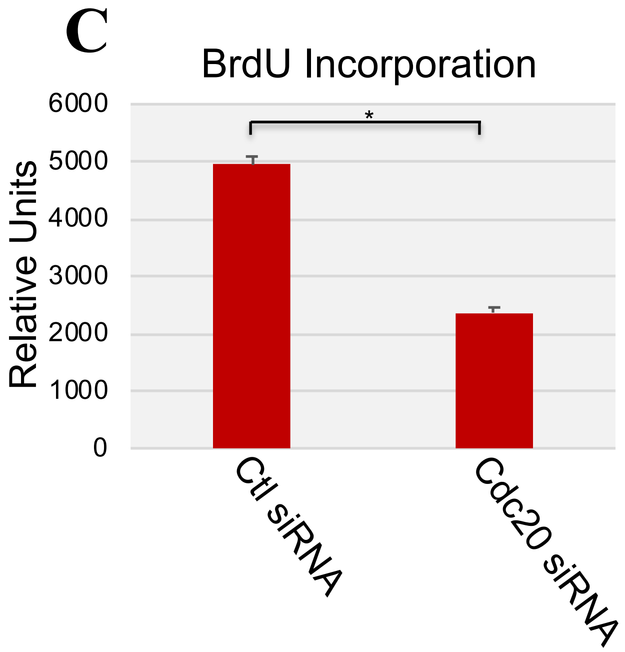

A

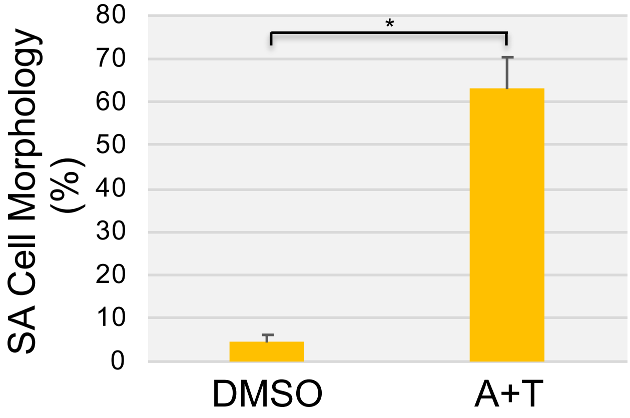
SA Cell Morphology (%)
80
70
60
50
40
30
20
10
0
*
DMSO
A+T

B

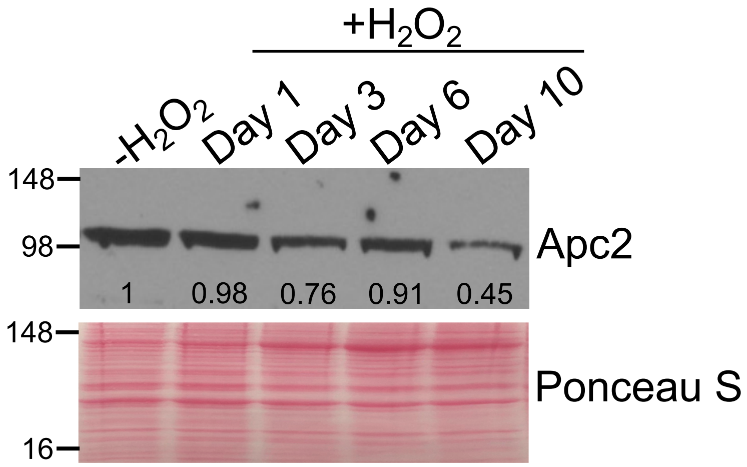
+H2O2
-H2O2
Day 1
Day 3
Day 6
Day 10
148
98
Apc2
1
0.98
0.76
0.91
0.45
148
16
Ponceau S

Volonte et al.,
Figure S5

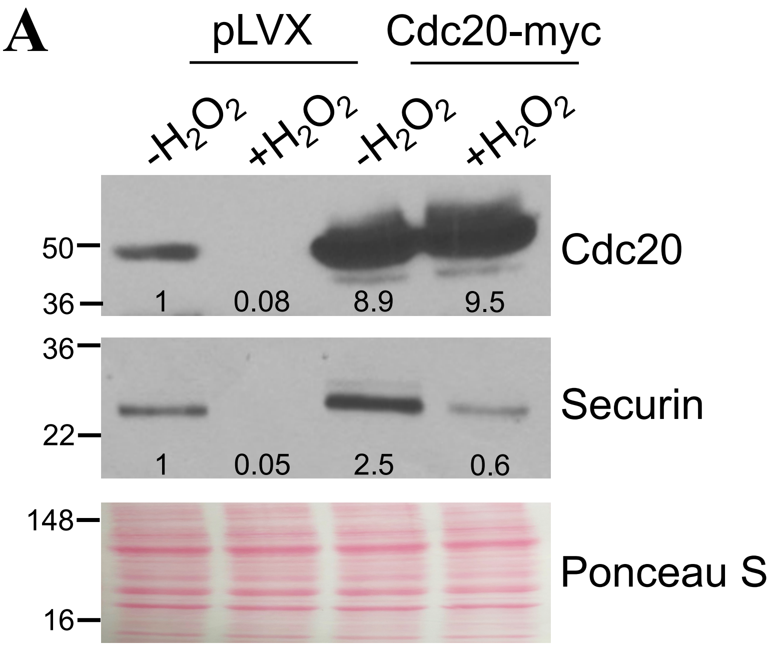

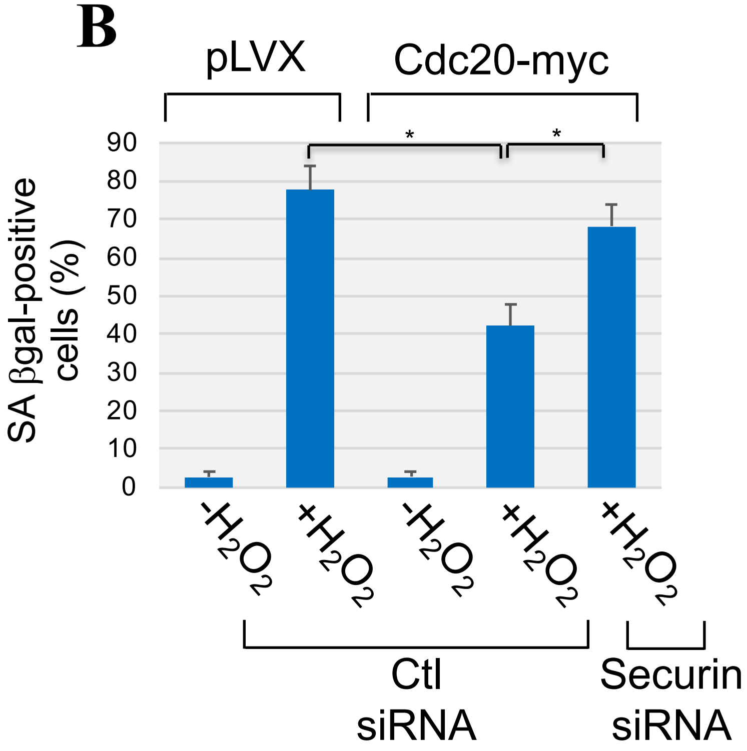

Volonte et al.,
Figure S6

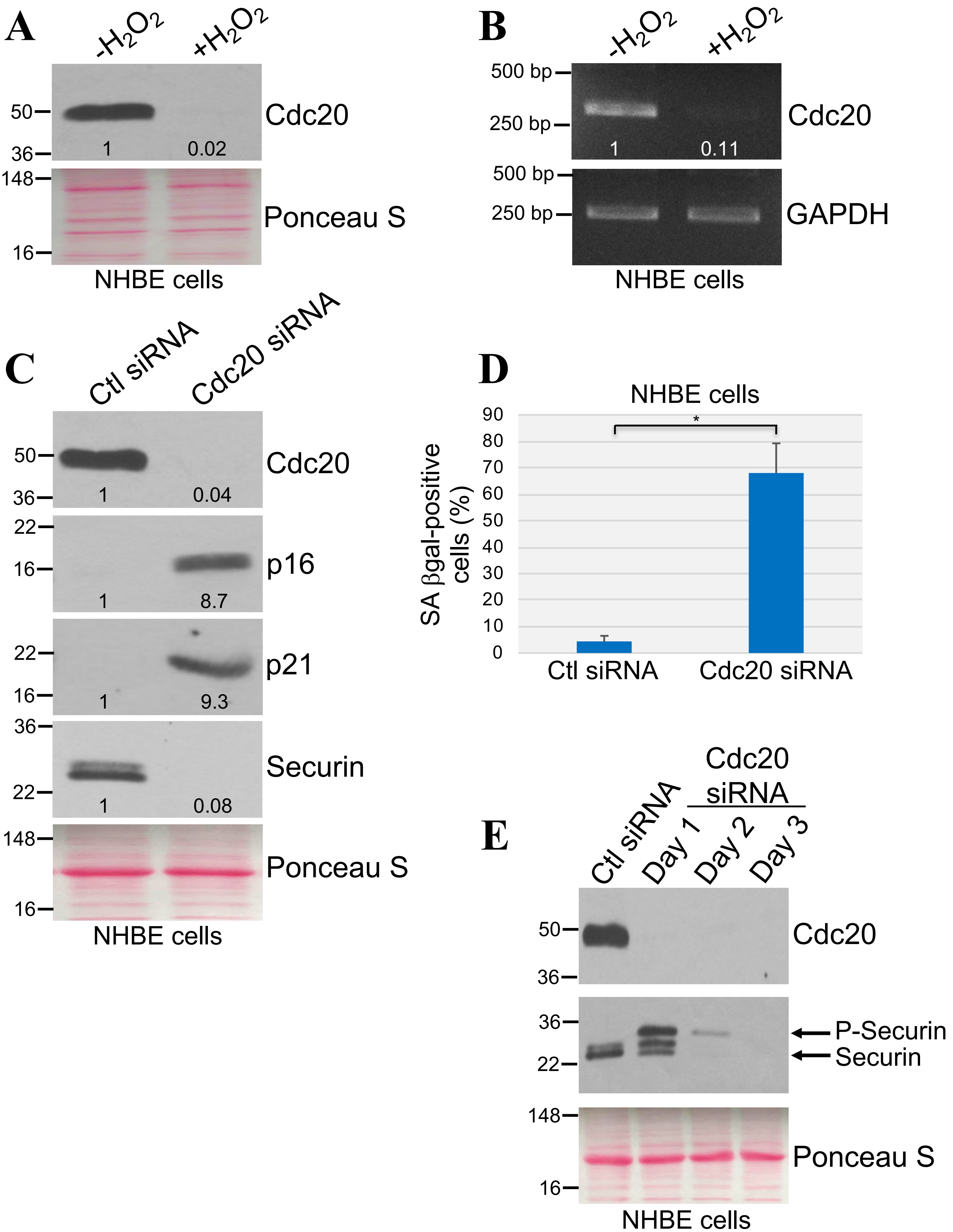

Volonte et al.,
Figure S7

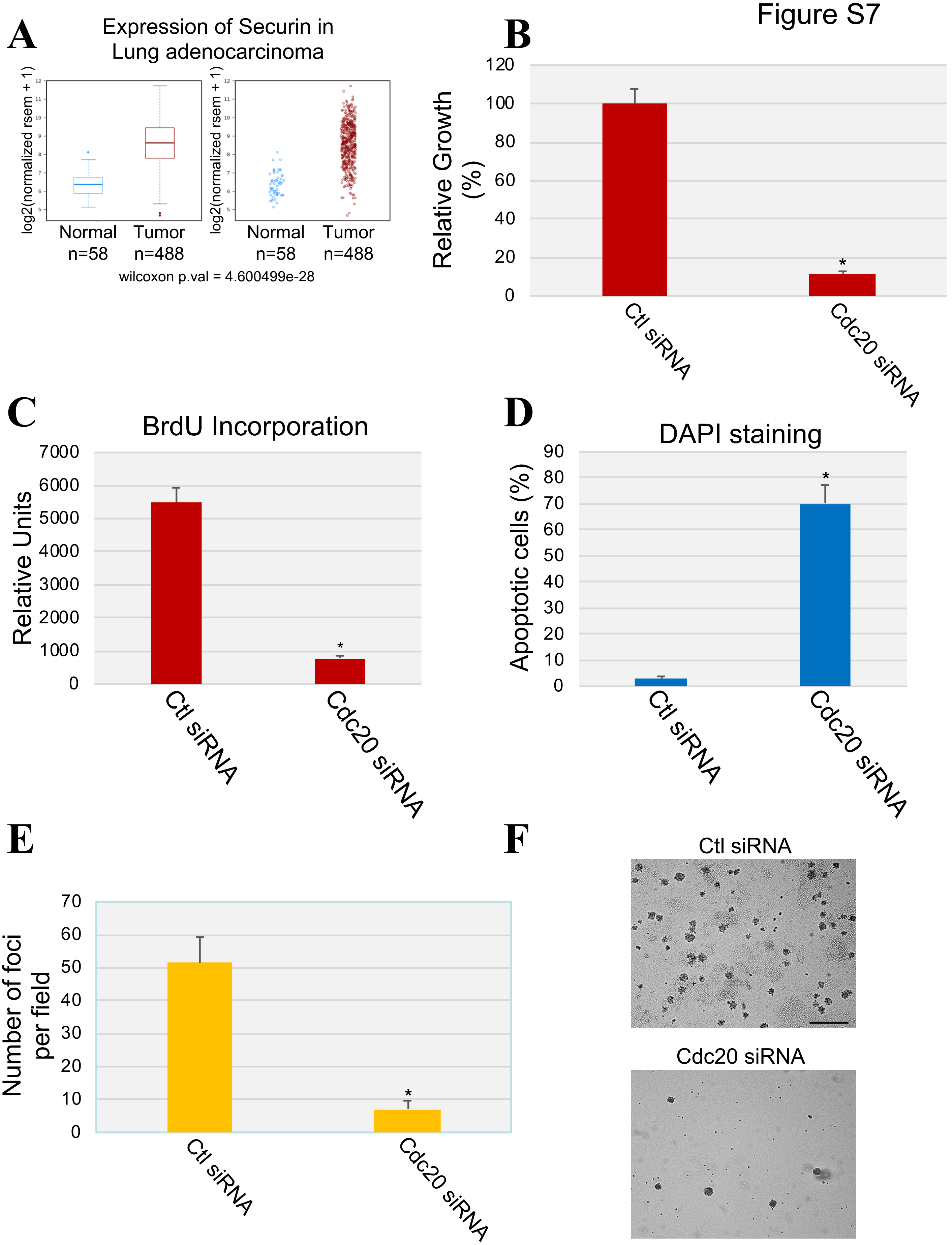

Volonte et al.,
Figure S8

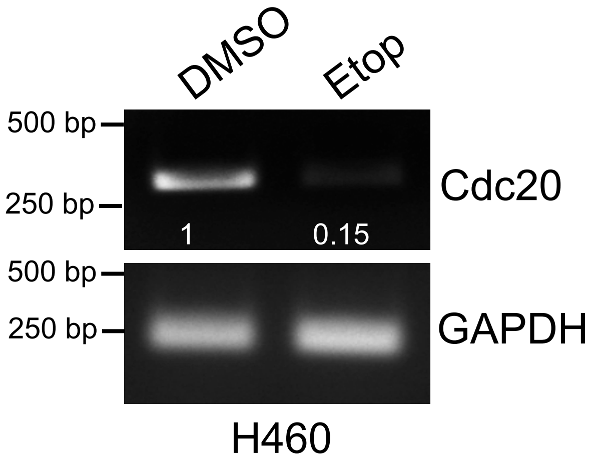

Volonte et al.,
Figure S9

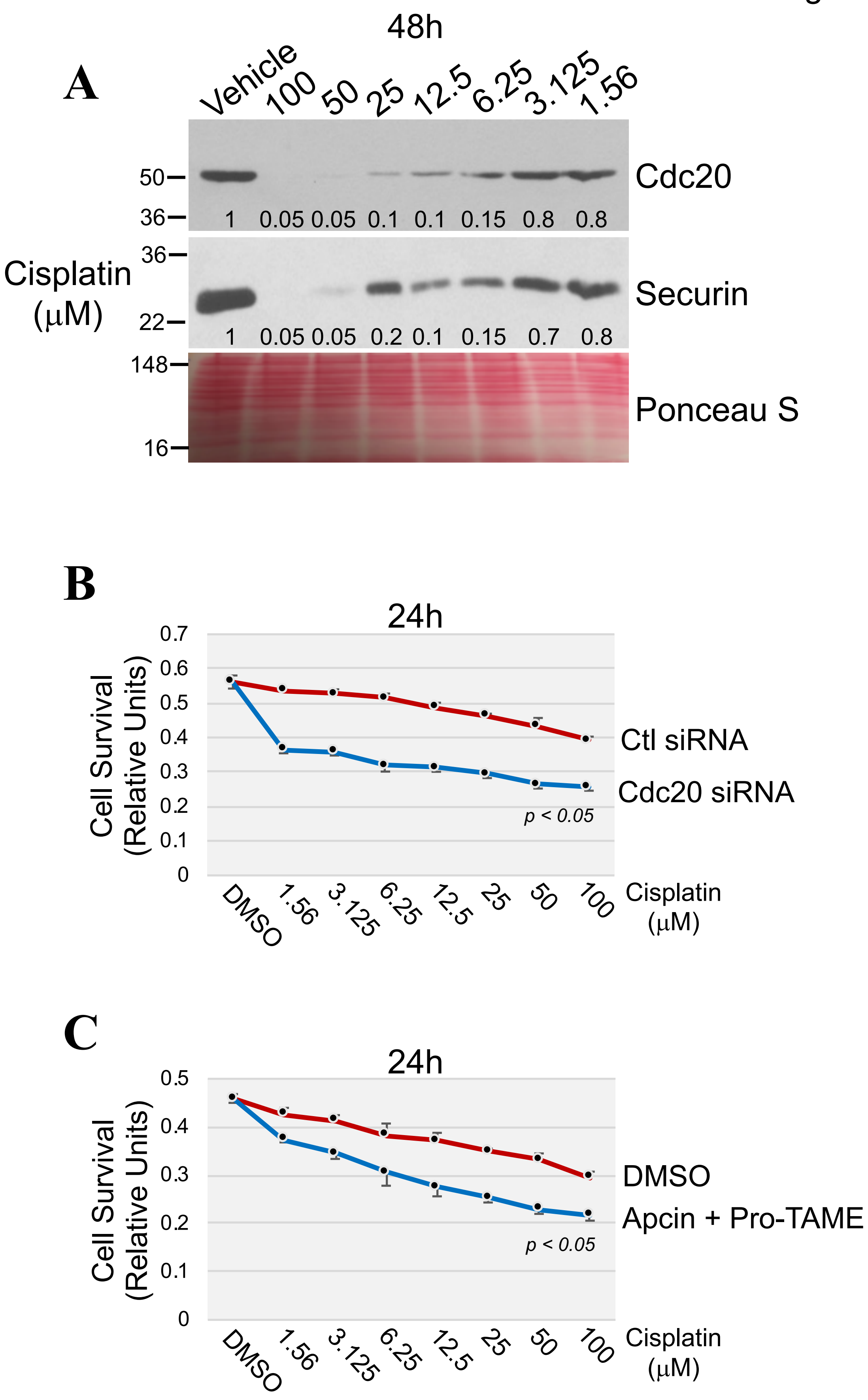

Volonte et al.,
Figure S10

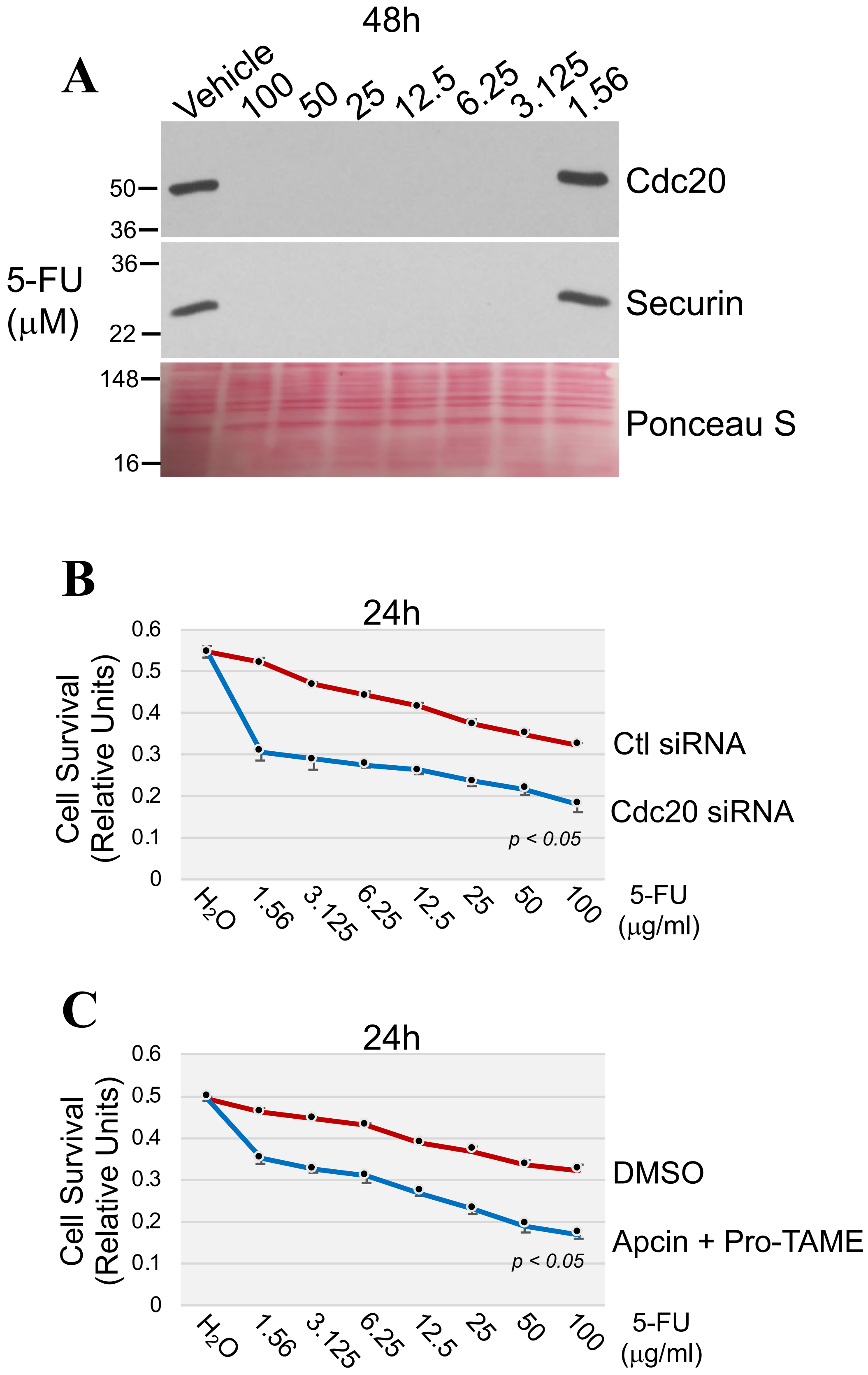

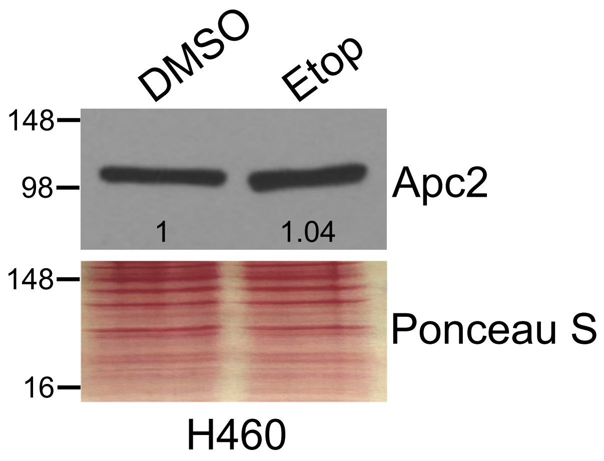
DMSO
Etop
148
98
Apc2
1
1.04
148
Ponceau S
16
H460

Volonte et al.,
Figure S12

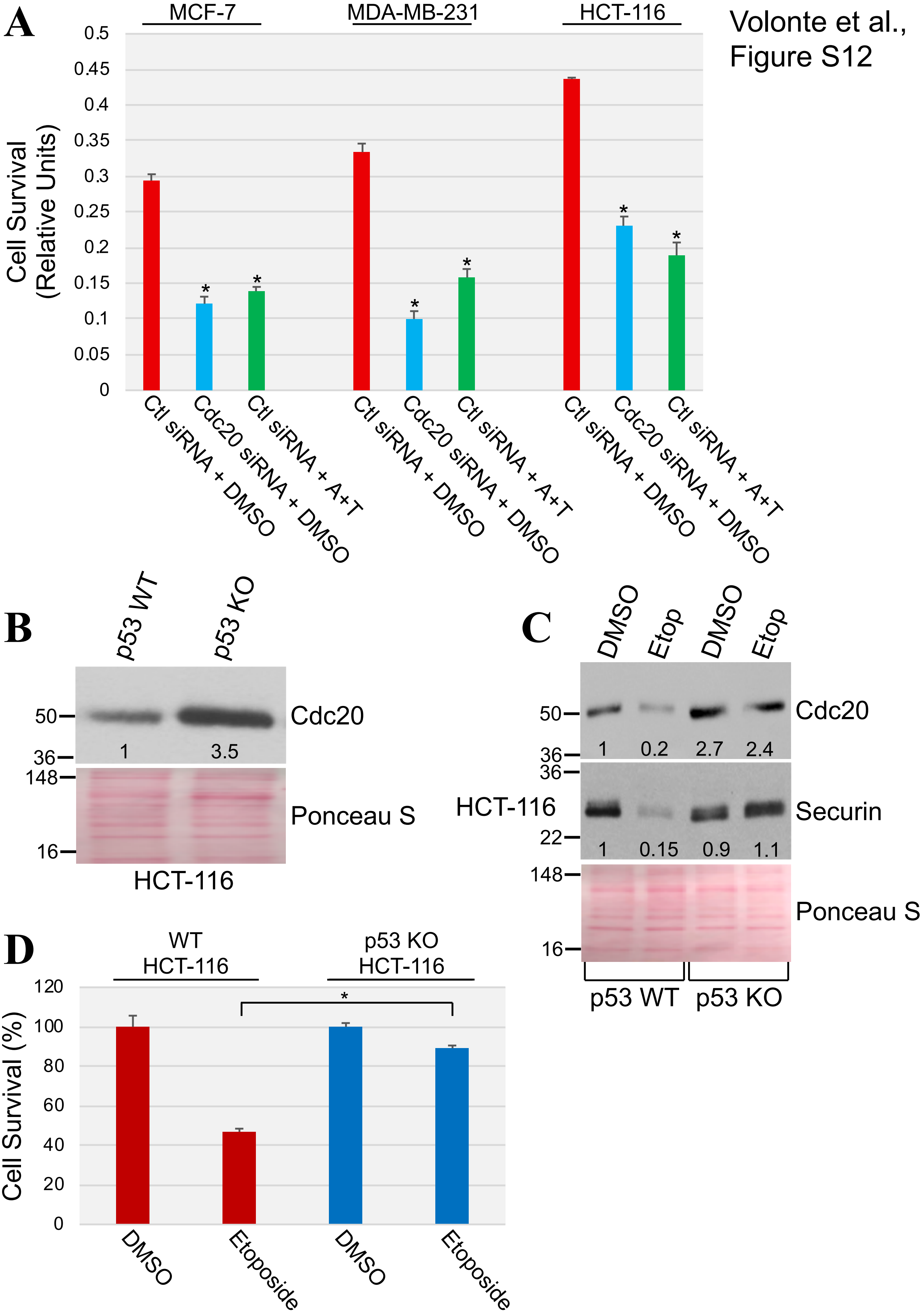

Volonte et al.,
Figure S13

A

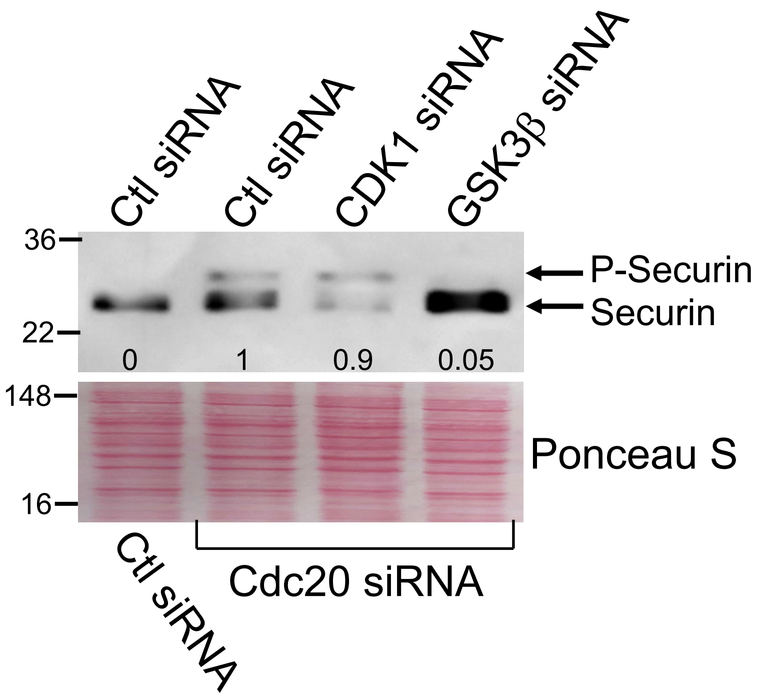

B

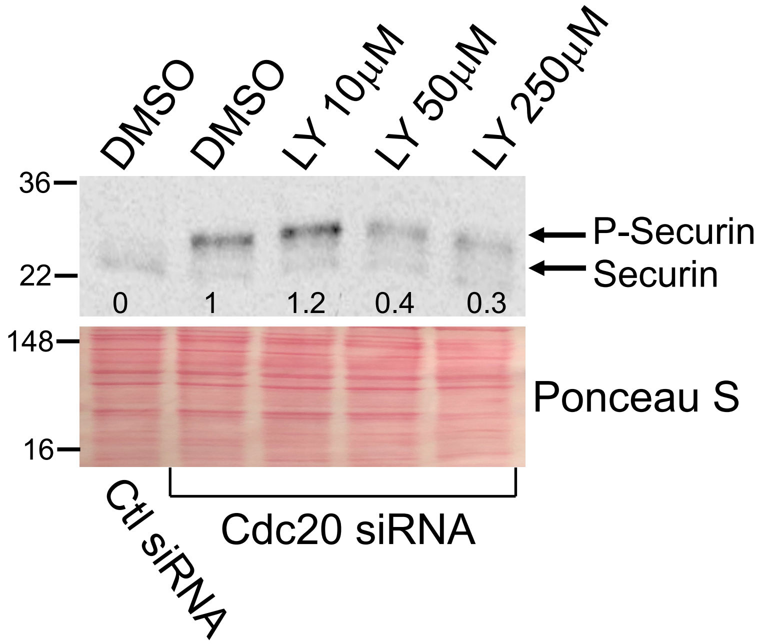

Volonte et al.,
Figure S14

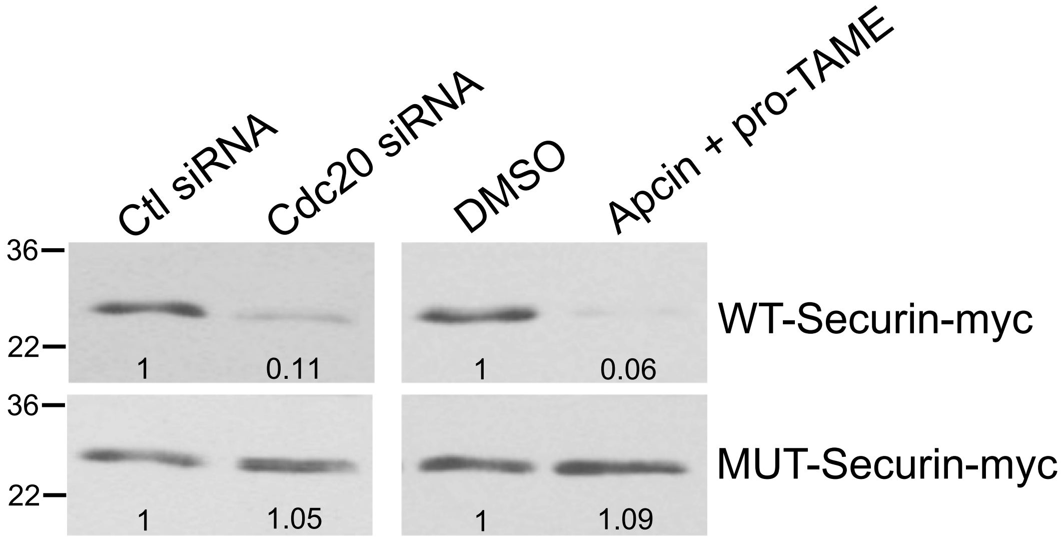

Volonte et al.,
Figure S15

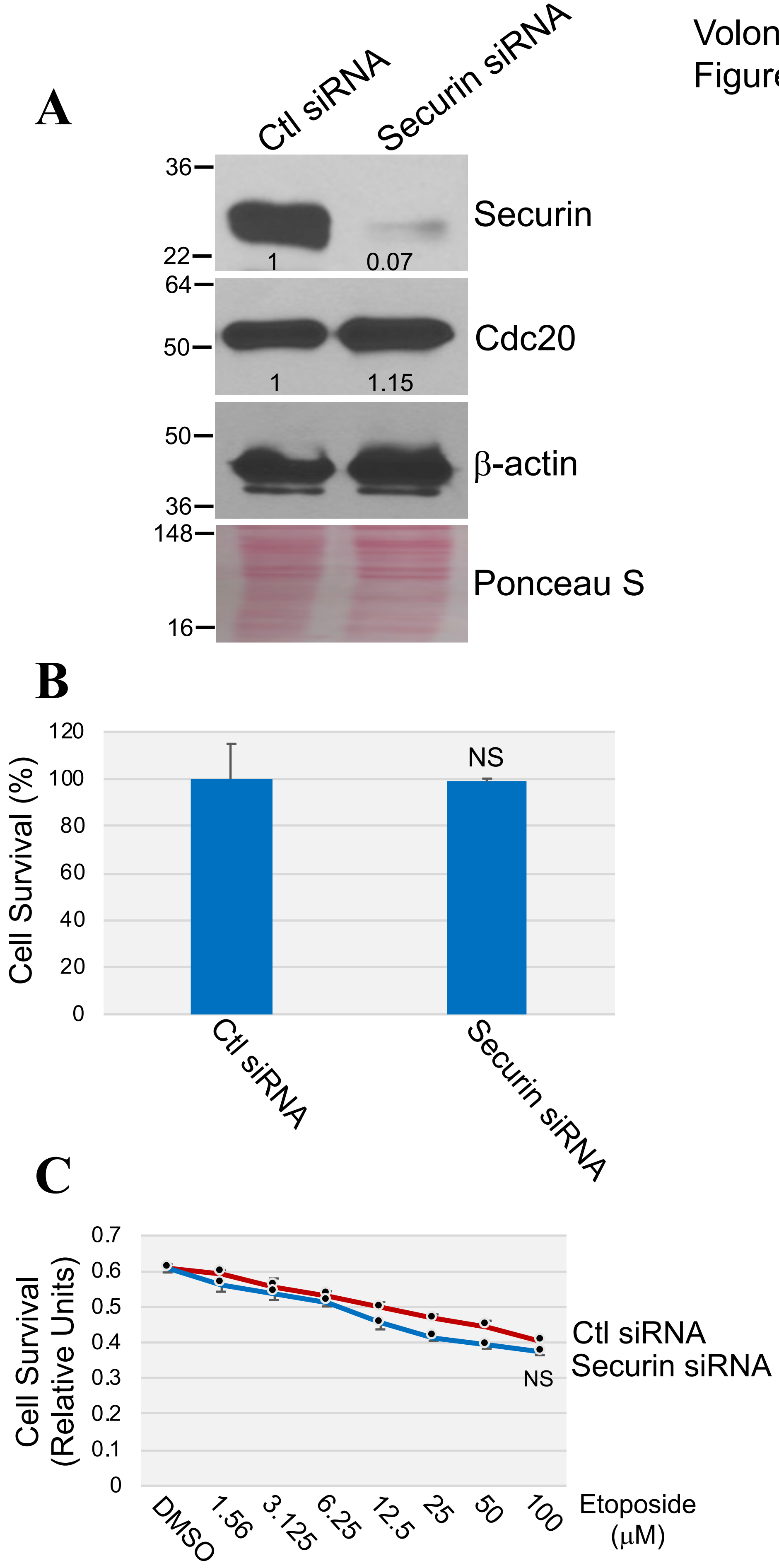

Supplement: Supplementary Figure [file mmc1.pdf]
